# LEARNet: A Learning Entropy-Aware Representation Network for Educational Video Understanding

**DOI:** 10.3390/e28010003

**Published:** 2025-12-19

**Authors:** Chitrakala S, Nivedha V V, Niranjana S R

**Affiliations:** Department of Computer Science and Engineering, CEG Campus, Anna University, Chennai 600025, India; chitrakala.au@gmail.com (C.S.); niranjanasr383@gmail.com (N.S.R.)

**Keywords:** entropy-aware learning, information bottleneck, entropy reduction, educational video understanding, semantic annotation, benchmark dataset

## Abstract

Educational videos contain long periods of visual redundancy, where only a few frames convey meaningful instructional information. Conventional video models, which are designed for dynamic scenes, often fail to capture these subtle pedagogical transitions. We introduce LEARNet, an entropy-aware framework that models educational video understanding as the extraction of high-information instructional content from low-entropy visual streams. LEARNet combines a Temporal Information Bottleneck (TIB) for selecting pedagogically significant keyframes with a Spatial–Semantic Decoder (SSD) that produces fine-grained annotations refined through a proposed Relational Consistency Verification Network (RCVN). This architecture enables the construction of EVUD-2M, a large-scale benchmark with multi-level semantic labels for diverse instructional formats. LEARNet achieves substantial redundancy reduction (70.2%) while maintaining high annotation fidelity (F1 = 0.89, mAP@50 = 0.88). Grounded in information-theoretic principles, LEARNet provides a scalable foundation for tasks such as lecture indexing, visual content summarization, and multimodal learning analytics.

## 1. Introduction

The widespread adoption of Massive Open Online Courses (MOOCs) and digital lecture libraries has highlighted a critical challenge: despite high enrolment, completion rates remain low [1], largely due to the inability of learners to efficiently locate key concepts within lengthy videos. While shorter videos improve completion, the comprehensive nature of educational content often necessitates longer formats. Traditional aids such as transcripts and manual timestamps offer limited assistance, they fail to provide the semantically rich, interactive experience necessary for effective learning. Analyzing educational content [2] particularly remains challenging due to the domain shift: instructional content is often static, symmetry-rich, and structured, with subtle pedagogical transitions that differ fundamentally from the dynamic, natural scenes typically targeted by existing video analysis methods. This work specifically targets static pedagogical visuals (slides, whiteboard, digital writing) that form the core of structured educational instruction. Dynamic demonstration-based videos such as laboratory experiments or mechanical operations fall outside the scope of this work, as they require motion-centric modeling pipelines and differ fundamentally from slide-centric teaching environments.

A primary problem is the profound domain shift. From an information-theoretic perspective, educational videos pose a severe spatial-temporal entropy problem: visually redundant frames can obscure the sparse but critical pedagogical information. State-of-the-art video models, trained on sports or surveillance footage, struggle to handle the prolonged visual stability and subtle, instructionally significant transitions characteristic of lecture media. In this context, even minor changes—such as a new equation on a slide or a diagram drawn on a board—can carry substantial instructional weight despite minimal pixel-level variation. Consequently, methods relying on motion cues (e.g., optical flow) or structural similarity (e.g., SSIM) often fragment the educational narrative and overlook the most meaningful moments.

Compounding this methodological deficit is severe data scarcity. Developing robust educational video understanding systems has been hindered by the absence of large-scale, fine-grained benchmarks. Manual annotation is immensely labor-intensive, and although large multimodal models (e.g., GPT-4o [3]) show promise, their computational demands and closed-source nature limit reproducible, foundational research. Similarly, while vision-language models (VLMs) like OWL-ViT enable open-world detection, they often produce spatially imprecise annotations, failing to capture the precise boundaries of educational elements like formulas, handwritten text, diagrams, speaker head.

From an information-theoretic perspective, this challenge can be framed in terms of Shannon’s entropy, which measures uncertainty or information content in a data stream. Educational videos exhibit high visual entropy due to static backgrounds and repetitive frames, yet low semantic entropy, as only a small subset of frames carry meaningful instructional information. The essential objective, therefore, is to design a system that minimizes redundant information while preserving maximal semantic content—an operational realization of Shannon’s principle of efficient information transfer.

**Figure 1:**
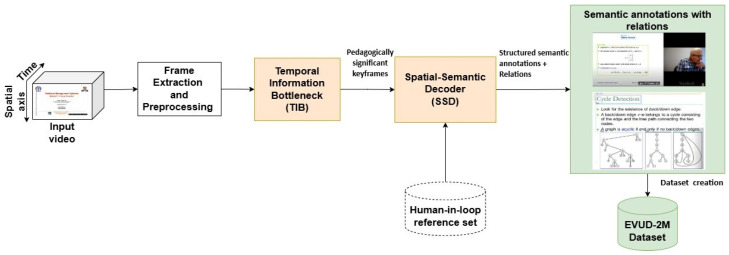
Overall architecture of the proposed LEARNet framework showing entropy-aware information flow from a high-entropy raw educational video to the formation low-entropy semantic graph formation and annotation.

To address these interconnected challenges, we introduce LEARNet (Learning Entropy-Aware Representation Network)—a deep learning architecture that integrates entropy-based filtering and semantic decoding to efficiently annotate structured educational video content semantically as described in Figure 1. LEARNet operationalizes Shannon’s principle of efficient information transfer by minimizing redundant visual entropy while retaining pedagogically significant content. Our work frames educational video understanding as a task of pedagogical information extraction and makes three key contributions:

1. A Novel Formulation of Instructional Structure: We identify an Instructional Steady-State in educational videos, characterized by long periods of visual consistency interrupted by brief, semantically rich transitions. Capturing this structure requires selecting frames that preserve pedagogically significant content, rather than relying on low-level change detection.
2. The Domain-Specific LEARNet Framework: We propose LEARNet, a deep learning architecture tailored to educational videos that integrates two novel components:
  - Temporal Information Bottleneck (TIB): Filters redundant frames using multi-modal cues to detect meaningful transitions and preserves only pedagogical significant frames.
  - Spatial-Semantic Decoder (SSD): Annotates these keyframes by constructing high-fidelity semantic graphs, capturing both the spatial layout and the semantic relationships among educational components, such as slides, diagrams, formulas, and titles. The Relational Consistency Verification Network (RCVN) ensures that these semantic relationships are coherent, minimizing errors and preserving instructional meaning.
3. EVUD-2M Benchmark: We introduce EVUD-2M, a two-million-frame educational video dataset generated by LEARNet. Starting from a 200-frame reference set, LEARNet applied a one-shot, semi-automatic annotation pipeline to produce fine-grained, multi-tier annotations for slides, diagrams, equations, and handwritten text. EVUD-2M integrates diverse sources and instructional formats, providing a scalable, robust benchmark for hierarchical educational video understanding and demonstrating LEARNet’s effectiveness as a domain-specific, entropy-aware annotation framework.

LEARNet is tailored for structured instructional content typically seen in lecture videos, such as slide presentations, digital whiteboards, and traditional blackboard teaching. The framework effectively manages instructor-overlaid content and gradual visual changes, emphasizing pedagogical settings where educational visuals remain largely stable to ensure clear knowledge delivery. Scenarios with high visual dynamics—like multi-person classroom interactions, group activities, or lab demonstrations—fall outside the current scope and present valuable opportunities for future exploration. While LEARNet is designed to leverage visual pedagogical cues, lectures where explanations rely primarily on audio may contain instructional transitions not visually apparent, motivating future multimodal (audio–text–vision) integration to capture richer educational context.

Extensive experiments show that LEARNet significantly outperforms existing approaches, reducing temporal redundancy by over 70% while achieving state-of-the-art annotation accuracy (mAP@50: 0.88). By providing both a novel, domain-specific framework and a large-scale benchmark, this work lays a solid foundation for downstream educational applications, including content summarization, visual question answering, multi-modal table of content generation and automated lecture structuring.

## 2. Literature Survey

The rapid expansion of digital educational content has created a persistent need for intelligent video understanding systems. However, progress in this area has been deprived by a fundamental mismatch: the core computational tasks of temporal segmentation (keyframe extraction) and spatial understanding (semantic annotation) have been dominated by the methods designed for dynamic, natural scenes, leaving the unique characteristics of instructional content largely unaddressed. This survey reviews the state-of-the-art, focusing on their collective failure to model the Instructional Steady-State.

### 2.1. Keyframe Extraction Techniques: A Paradigm Ill-Suited for Educational Content

Keyframe extraction is the process of selecting a minimal, non-redundant subset of frames that best represent the core semantics of a video sequence. For educational videos, which are often characterized by static scenes, subtle content changes, and a high density of informational elements, this task poses unique challenges. Existing keyframe extraction methods can be broadly classified into three categories based on their feature extraction methodology.

#### 2.1.1. High-Entropy Feature Methods

Pixel-based [1] and optical flow approaches are highly effective at detecting sharp cuts and motion—high-energy signals. However, in educational videos, the most information-rich events—such as a new equation on a static slide—may involve minimal visual change [4,5,6,7,8]. Recent adaptive methods [9,10,11,12,13] improve efficiency but remain dependent on low-level metrics like sharpness or luminance, failing to capture conceptually important transitions.

#### 2.1.2. Mid-Level Feature Methods

Techniques leveraging intermediate representations such as Histogram of Oriented Gradients (HoG) [14] or Convolutional Neural Network (CNN) [15] features, offer improved robustness over raw pixels. Some combine these with structural similarity metrics [16] or SVM classifiers [17,18] for keyframe selection. While effective at detecting overall visual changes, these methods remain trapped in a “semantic gap,” missing fine-grained in-slide changes that carry significant pedagogical value. For instance, SIFT-based clustering [19] excels in distinguishing overall scene composition but is blind to fine-grained, in-slide evolutions of a diagram that constitute meaningful information. Template-matching approaches [20] are often constrained to predefined action sets, limiting their adaptability to the diverse and often static formats of educational videos.

#### 2.1.3. Semantic and the Information-Theoretic Methods

Semantic-based methods recognize the need for content-awareness. Early works highlighted this need without providing computational models [21], while later approaches used coarse proxies like slide transitions [22] or integrated transcripts and visual cues [23,24] for temporal segmentation but lack fine-grained visual reasoning, often missing non-textual elements like diagrams and figures. Multimodal approaches [25,26,27,28] represent the most advanced step, fusing audio, text, and vision. However, they typically produce topic-level segments, failing to pinpoint the exact keyframe where a new concept is visually introduced. They remain indirect, often relying on user comments [29] or silence detection [30], and lack the fine-grained visual reasoning to identify the specific frame where pedagogical semantics shift, thus failing to act as an optimal encoder for instructional content.

Recent entropy-driven approaches in both vision and non-vision domains further strengthen the theoretical basis of our entropy-aware framework. For instance, phase-entropy-based fault diagnosis models in rotating machinery have demonstrated strong capability in identifying subtle temporal transitions by quantifying dynamic uncertainty in physical signals, illustrating the general applicability of entropy-based change detection [31]. Recent entropy-based studies in video analysis further support our entropy-aware formulation. Entropy-driven Unsupervised Key point Representation Learning [32] in Videos demonstrates how local spatial entropy can identify stable visual structures across frames. SGE-Net [33] employs an information entropy map to enhance video object detection, highlighting high-information regions in dynamic scenes. Additionally, Shot Segmentation Based on Von Neumann Entropy for Key Frame Extraction uses Von Neumann entropy to detect shot boundaries and extract representative frames [34]. These works collectively show that entropy is a powerful tool for detecting meaningful visual transitions, directly reinforcing the foundations of our Instructional Steady-State and entropy-guided Temporal Information Bottleneck.

### 2.2. Semantic Annotation: A Fragmented Landscape Lacking Educational Focus

Annotation of educational videos faces similar limitations. Existing datasets and tools are often too narrow, coarse, or misaligned with educational needs, resulting in inconsistent or incomplete semantic understanding.

#### 2.2.1. Specialized but Static Datasets

Datasets like ChartQA [35], AI2D [36], and PlotQA [37] provide annotations for specific visual elements but are restricted to static images of charts and diagrams, missing the temporal dimension and the diversity of visual aids (tables, blackboards, handwritten text) present in real lecture videos. Traditional computer vision tools like HOG or OCR [38] captures basic elements but fail to provide the fine-grained, semantic classification with multi-label annotations necessary for understanding frames that contain multiple pedagogical components.

#### 2.2.2. Coarse Temporal Segmentations

Systems such as ATLAS [24] and other systems [21,27] offer segment-level annotations, sufficient for timeline-based Table of Contents. However, they operate at a rough granularity, completely lacking the object-level labels within frames necessary for detailed navigation and interaction, thus providing only a low-resolution semantic understanding.

#### 2.2.3. General-Purpose Multimodal Models

Large-scale multimodal datasets, such as MERLOT Reserve [39], How2 [40], and Ego4D [41] are misaligned with educational objectives. Their annotations are inclined towards general activity recognition or egocentric perception rather than the precise identification of academic elements like mathematical formulas or schematic diagrams.

#### 2.2.4. The Gap in Scalable, Finely Annotated Benchmarks

Beyond the models, the datasets themselves present a bottleneck. While several resources have been curated for lecture video understanding, they collectively lack the scale, granularity, or focus required for comprehensive semantic parsing. As summarized in Table 1, existing datasets are highly fragmented. Some, like the large-scale NPTEL Videos [42], provide vast amounts of raw content but lack any keyframe selection or fine-grained annotations, making them unsuitable for training sophisticated understanding models. Others are narrowly focused on a single task, such as slide segmentation (SPaSe [43], WiSe [44]) or text detection (LectureVideo DB [45]) but lack the diversity of annotations needed for holistic scene understanding. Furthermore, resources like ALV [46] are synthetic, lacking the authenticity of real classroom recordings, while multimodal datasets like Multimodal Lecture Presentations Dataset [47] prioritize content alignment over pixel-precise, region-level analysis. Lecture Bank [48] focuses solely on lecture slides and their prerequisite relations; no alignment with transcripts or focus on video processing; includes artificially generated lecture videos; lacks authentic educational contexts and excludes slide-based visual content. VLEngagement [49] examines engagement in lectures but does not delve into the structural or semantic understanding of video content.

As summarized in Table 1, existing datasets either provide raw videos without keyframe selection or focus narrowly on tasks like slide segmentation or text detection, leaving broader semantic understanding unaddressed. Synthetic datasets often lack authenticity, while multimodal resources emphasize content alignment over pixel-precise, region-level analysis. Collectively, there is no domain-specific benchmark that simultaneously offers: (1) large-scale, authentic data, (2) pedagogically meaningful keyframe selection, and (3) fine-grained, region-level semantic annotations.

#### 2.2.5. The Integration Gap in Foundational Models

Foundational models such as OWL-ViT [51,52], GLIP [53], Segment Anything Model (SAM) [54], Grounding DINO [55], DETR-50 [56] and ViLD [57] offer new capabilities in open-vocabulary detection but suffer from imprecise localization, dependency on precise text prompts and class-agnostic outputs. Despite advances in vision-language and segmentation models, no existing methods offer an end-to-end, domain-specific approach for generating accurate, structured semantic maps for annotating educational videos.

In summary, the literature exposes a recurring and critical issue: existing methods for keyframe extraction and semantic annotation are poorly suited for educational videos because they are entropy-blind. They struggle to handle the unique information transformation challenges inherent in instructional content, primarily because they:

- Rely on flawed temporal assumptions, emphasizing high-entropy cues like motion rather than detecting low-entropy but pedagogically significant changes, which leads to inefficient compression of instructional information.
- Annotate at inappropriate granularities, resulting in incomplete semantic decoding that misses concentrated, high-value educational content.
- Do not provide a domain-specific framework capable of optimizing information retention, leaving the potential of modern models underutilized.

No prior work offers a holistic approach that fully captures the hierarchical structure of educational videos—first segmenting content based on pedagogical intent, then parsing each segment into a structured, relational scene graph. This highlights a clear and pressing gap: the absence of a comprehensive, entropy-aware benchmark that simultaneously delivers (a) large-scale, authentic educational data, (b) pedagogically meaningful keyframe selection, and (c) fine-grained, region-level semantic annotations. To address this, we introduce the LEARNet architecture, grounded in information-theoretic principles, along with the EVUD-2M benchmark, designed to bridge this gap by tackling both the temporal and spatial challenges inherent in educational video understanding.

## 3. The EVUD-2M Benchmark: A Dataset for Hierarchical Educational Video Parsing

A major limitation in the field of educational video analysis is the absence of a large-scale and diverse dataset that captures the richness of different instructional formats. Existing benchmarks often fail to represent the temporal flow of lessons or the fine-grained spatial details that characterize teaching interactions. To address this gap, we introduce EVUD-2M, a comprehensive benchmark consisting of approximately two million annotated frames sourced from a wide variety of educational video formats—including black board lectures, lectures with slides, demonstrations, and digital whiteboard sessions. This dataset specifically focuses on undergraduate and postgraduate-level university instruction, as evidenced by the advanced curriculum of its primary sources like NPTEL, which covers specialized disciplines from Aerospace Engineering to Quantum Mechanics. This dataset is designed to support both temporal segmentation and detailed spatial parsing, offering a robust foundation for training and evaluating deep learning models. By enabling a better understanding of the timing, structure, and visual semantics of instructional content, EVUD-2M provides a realistic and scalable basis for advancing artificial intelligence in educational video comprehension and analysis.

### 3.1. Curation Strategy and Semantic Coverage

The EVUD-2M benchmark is designed to capture the broad visual diversity of higher-education instructional videos. Rather than focusing on a single format, it integrates multiple teaching styles—including slide presentations, classroom explanations, whiteboard and blackboard lectures, and digital writing sessions. The dataset is sourced predominantly from undergraduate and postgraduate courses, as reflected in the advanced topics covered in NPTEL recordings such as Aerospace Engineering, Quantum Mechanics, and Nanotechnology.

EVUD-2M spans a wide academic spectrum, covering STEM fields alongside Humanities, Social Sciences, and Management. This diversity exposes the model to varied presentation styles and knowledge densities, ranging from dense mathematical derivations to concept-driven, discursive lectures. As a result, the dataset closely mirrors higher-education environments and provides a robust foundation for models aiming to interpret instructional visuals across formats.

The benchmark intentionally focuses on static pedagogical content—slides, written annotations, diagrams, and board work—where visual structure carries core instructional meaning. In contrast, demonstration-based or vocational videos (e.g., laboratory procedures, mechanical operations) are currently under-represented because they require motion-centric modeling rather than static visual analysis. Extending EVUD-2M to include such dynamic instructional videos is part of planned future work.

Given this focus, EVUD-2M is best suited for vision-centric frameworks such as LEARNet, which rely on static visual cues to detect instructional transitions. Videos where critical information is conveyed primarily through audio further motivate the need for future multimodal integration.

- Authentic Lecture Context: The benchmark draws primarily from the NPTEL and ClassX repositories, offering a broad and realistic collection of lecture videos. This ensures models are exposed to the inherent challenges of the Instructional Steady-State, including static layouts, subtle transitions, and a mix of visual aids.
- Preventing Layout Bias: To avoid overfitting to specific video styles, a large set of standalone slides from SlideShare-1M is included. This helps models focus on understanding the actual meaning within slides, diagrams, and tables, instead of depending on video layout or recording patterns.
- Targeted Component Enhancement: Additional data on figures and tables is incorporated to strengthen model learning on key visual instructional elements. This enrichment supports fine-grained, multi-label annotations and enables precise scene parsing aligned with the goals of the LEARNet framework.

To further clarify the educational scope and context of EVUD-2M, Table 2 provides a detailed breakdown of the educational stages, content types, and representative examples included in the benchmark, directly addressing the reviewer’s query on model applicability.

### 3.2. Dataset Curation and Composition

Building on this foundation, the curation of EVUD-2M was guided by a clear strategy to ensure both diversity and depth. We sourced content from a wide array of authentic educational materials. The dataset was curated through a multi-source strategy to ensure comprehensive coverage of academic visual aids and presentation styles, as detailed in Table 3.

The dataset integrates content from three primary educational sources: 19,578 NPTEL videos preserving original disciplinary distributions, ClassX lecture materials from 21 Stanford courses, and 191,000 representative slides from the SlideShare-1M corpus.

To build a diverse educational video corpus, we curated material from NPTEL, ClassX, and SlideShare, covering 25 subject domains and about 550 courses, totaling over 18,000 h of instructional content. From this collection, 200 manually annotated seed keyframes were created and expanded—via the entropy-guided SSD–RCVN pipeline—into more than 2 million high-quality annotated keyframes, forming the final EVUD-2M benchmark. A critical innovation in EVUD-2M’s construction is the application of our Temporal Information Bottleneck (TIB) for semantic keyframe selection. This process distilled 949,000 pedagogically meaningful keyframes from an initial pool of 2,000,000 traditional I-frames, achieving a 52.5% volume reduction while preserving essential educational content. Finally, to ensure robust coverage of educational graphics, we incorporated auxiliary datasets, including TableBank [58], STDW [59] for scientific diagrams, and curated images from DuckDuckGo images [60], for diverse visual representations. This multi-source approach ensures diversity across institutional styles, presentation formats, and academic disciplines. To prevent bias toward specific visual templates, we implemented strategic sampling—selecting 1–5 representative slides per presentation deck—effectively mitigating layout overfitting while maintaining content richness. The resulting dataset maintains balanced representation across core educational formats: slide-based presentations (77.2%), blackboard instruction (13.1%), and digital writing interfaces (9.7%), establishing a comprehensive foundation for developing and evaluating educational video understanding systems.

**Table 3:** Composition of the EVUD-2M Benchmark.

Dataset Name	Content Type	Key Statistics	Usage in Work
NPTEL [42]	Educational lecture videos	733K keyframes 19,578 videos Sampled from 27 disciplines	Primary source for authentic, diverse lecture content across STEM and humanities.
ClassX [50]	Lecture video clips	25K keyframes 21 Stanford courses 258 query slides and 8619 training slides	Supplements with varied lecture styles and institutional presentation formats.
SlideShare-1M [61]	Presentation slides	191K slides (curated subset of 977K slides) Sampled from 31,923 slide decks	Provides high-quality, standalone slides to prevent model overfitting to specific video layouts.
Tablenet [62], TableBank [56]	Scanned document images	Derived from Marmot/ICDAR 2013 datasets	Enables robust detection of specific educational components (tables, plots, diagrams) through targeted training
Roboflow Platform [63]	Computer vision datasets	Access to 200,000+ datasets 50,000+ pre-trained models	

### 3.3. Comparison with State-of-the-Art Educational Video Datasets

Table 4 provides a systematic comparison positioning EVUD-2M within the landscape of video and image datasets. While general video datasets like TVQA [64], HowTo100M [65], and COIN [66] focus on dynamic content with limited visual annotations, and specialized educational systems like LectureNet [67] and EduNet [68] offer basic keyframe extraction without detailed region-level labels, EVUD-2M addresses the critical gap for comprehensive educational video understanding. Its foundational integration of time and context sets EVUD-2M apart from static diagram resources such as AI2D and ChartQA that lack temporal dimension. It is built not just to recognize educational elements, but to understand their sequence and relationship within a live lesson.

Distinguishing Features and Applications: EVUD-2M introduces several pivotal capabilities that collectively advance educational video analysis:

- Intelligent frame selection: Instead of sampling frames at fixed intervals, we employ a pedagogical significance scorer that identifies the most instructionally critical moments.
- Educational Element Annotation: The benchmark provides detailed, region-level labels for academic components like equations and diagrams, moving beyond generic object detection.
- Inherent Generalization: By leveraging modern vision-language models, the dataset equips AI systems with zero-shot capabilities, allowing them to recognize educational concepts they were not explicitly trained on.
- Unified multi-format Analysis: It seamlessly handles the full spectrum of instruction—from slides and digital whiteboards to traditional blackboards—within a single, coherent framework.

This design directly supports practical applications, including automated content indexing, lesson summarization, and visual question-answering systems.

Scalability and extensibility: The long-term utility and extensibility of the EVUD-2M benchmark are anchored in a deliberately scalable architecture. A pivotal innovation is our highly efficient annotation framework, which enabled the propagation of high-quality labels across the entire corpus of two million frames from a minimal set of only 200 manually annotated references. The reliability of this semi-automatic strategy, which operates at a 10,000:1 ratio, was rigorously assessed. A stratified validation subset was subjected to a manual audit by three independent annotators, who achieved a strong inter-annotator agreement (Cohen’s κ = 0.84). This human evaluation confirmed a 93.2% annotation accuracy rate, with the limited errors predominantly confined to low-resolution board content. This supports the scalability of the semi-automatic 10,000:1 annotation strategy. This robust validation, coupled with the dataset’s inherent modularity, ensures that the benchmark can be continuously expanded and adapted to emerging educational formats while maintaining consistent annotation integrity.

### 3.4. Data Preprocessing for Robust Parsing

A standardized preprocessing pipeline was applied to ensure consistency across all videos while preserving the semantic integrity of instructional content:

1. Frame Normalization and Quality Enhancement: Video frames were standardized and resized to the resolutions required by the downstream modules. Adaptive filtering was applied to reduce compression artifacts and enhance clarity without altering pedagogical meaning.
2. Illumination Normalization: Histogram equalization and gamma correction were used to stabilize brightness and contrast, improving robustness to varied classroom lighting and recording setups.
3. Semantic-Preserving Augmentation: Light augmentations such as mild rotations, flips, and subtle color adjustments were introduced to improve generalization. Only transformations that preserved the semantic interpretation of diagrams, text regions, and other instructional elements were applied.

These preprocessing steps ensure that EVUD-2M is a carefully prepared benchmark that supports reliable parsing, robust feature extraction, and semantically meaningful educational video understanding.

### 3.5. Multilevel Annotation Framework

The EVUD-2M benchmark features a Multilevel Annotation Framework co-designed with the LEARNet architecture, enabling a direct transition from raw video frames to structured educational understanding. The framework consists of three integrated tiers:

- Image-Level Filtering: Frames are first categorized to separate pedagogically relevant scenes (e.g., slides, board content) from non-informative visuals (e.g., blank screens, logos), ensuring computational effort is focused on meaningful instructional content.
- Region-Level Spatial Annotation: Pixel-accurate segmentation masks are provided for nine educational element categories—including text blocks, diagrams, equations, and titles—supporting downstream tasks such as OCR, diagram parsing, and fine-grained content retrieval.
- Relational-Level Semantic Graphing: Triplet-based annotations (e.g., <figure, has, figure_title>) model relationships between components, capturing instructional structure and linking visual elements with their explanatory context.

Together, these tiers give LEARNet the structured guidance it needs to reliably parse educational videos and understand their higher-level meaning.

### 3.6. Annotation Scaling and Verification

To handle nearly two million images in the EVUD-2M benchmark, we needed a semantic labeling model that is fast yet accurate with relational consistency verification. We started with 200 carefully chosen example frames as seed set, then used a unified model built upon LEARNet to spread labels automatically through the rest of the dataset. First, a basic version of LEARNet learned from those examples; subsequently. First, a system examines through videos, retaining just the pedagogically significant keyframes to cut down on what needs detailed examination. Then, another part analyzes those selected images, creating automatic labels in multi-level annotation framework. To ensure accuracy without excessive work, we double-checked the labels only when the system remained very sure about them—specifically, under an 80% confidence level. Furthermore, a random 5% audit was conducted on all high-confidence annotations to ensure bias was not propagated. This methodology ensured the benchmark’s reliability while maintaining an efficient annotation ratio of approximately 10,000:1 (unlabeled to manually verified frames).

## 4. Methodology

This section introduces LEARNet (Learning Entropy-Aware Representation Network), a framework developed to tackle the fundamental challenges of understanding and structuring information in educational videos.

### 4.1. Overall Method

**Figure 2:**
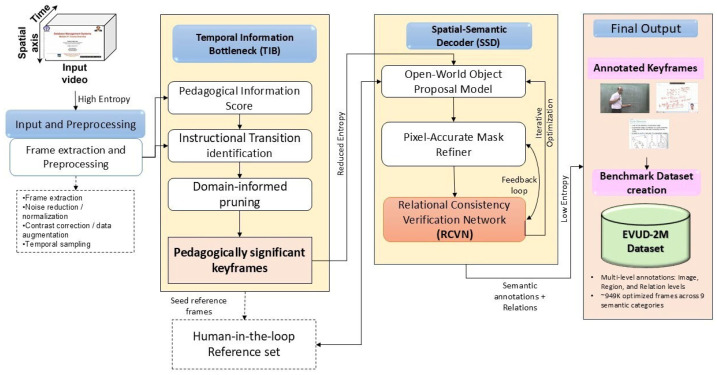
LEARNet: Learning Entropy-Aware Representation Network: High-to-low entropy transformation via Temporal Information Bottleneck and Spatial–Semantic Decoder.

LEARNet is a two-stage, entropy-aware framework designed to convert a high-entropy educational video stream into a low-entropy semantic representation aligned with instructional structure. As shown in Figure 2, the model operates through (1) pedagogically grounded keyframe segmentation and (2) fine-grained semantic decoding with relational consistency verification. Our system is developed under the assumption of visually stable instructional settings, where slide-based, whiteboard, and digital writing interfaces dominate, and does not address high-motion environments such as group discussions or lab demonstrations.

The Temporal Information Bottleneck (TIB) identifies pedagogically meaningful transitions by distinguishing visually obvious changes from those that carry genuine instructional value as described in Algorithm 1. It acts as an adaptive filter, retaining only frames where the teaching context meaningfully shifts. TIB is driven by a learned Pedagogical Information Score (PIS) that combines three cues:

- Structural Consistency Change to detect scene transitions,
- Domain-Weighted Saliency to emphasize educationally important regions, and
- Semantic Cluster Coherence to ensure each keyframe represents a distinct instructional unit.

Rather than selecting frames based on visual difference, TIB prioritizes frames that are instructionally consequential. A final domain-informed pruning step removes non-informative content (e.g., blank slides, static speaker shots), producing a compact yet semantically complete set of keyframes. Algorithm 1 summarizes the full procedure.
**Algorithm 1:** Temporal Information Bottleneck (TIB)1:**Input:** Sequence of video frames *F = {f*_1_*, f*_2_*, …, f_n_}* frame rate *fps*2:**Output:** Pedagogically significant keyframes *K = {k*_1_*, k*_2_*, …, k_r_}*, moderate significance frames *K’ = {k’*_1_*, k’*_2_*, …, k’_s_}*3:**// Step 1:** Pedagogical Information Scoring4:**for** *i* ← *2 to n* **do**5:      Compute *MSEᵢ* ← *$\frac{1}{N}\sum_{k=1}^{N}{\left(I_{i}\left(k\right)-I_{i-1}\left(k\right)\right)}^{2}$// structural saliency*6:      Compute *SaliencyMapᵢ* ← *ComputeSaliencyMap(fᵢ)*7:      Compute *SaliencyValueᵢ* ← *Mean (SaliencyMapᵢ) // content saliency*8:      Set weight *α* ← *0.67*9:      Compute *SignificanceScoreᵢ* ← *α × MSEᵢ + (1 − α) × SaliencyValue_i_//pedagogical information score*10:**end for**11://**Step 2:** Instructional Transition IdentificationApply *Clusters* ← *DensityPeakClustering({SignificanceScore*_2_*, …, SignificanceScore_n_})*12:Initialize candidate keyframes: *K* ← *{$f_{c}$|$f_{c}$ ∈ Clusters.Centroids}*13:**// Step 2:** Domain-informed pruning14:*Initialize K’ ←∅*15:**for each** *frame k∈ K* **do**16:      Compute *duration* ← *ComputeFrameDuration(k, fps)*17:      Compute *personArea* ← *ComputePersonArea(k)*18:      Compute *ssim* ← *ComputeSSIM (k, previousKeyframe)*19:      Compute *saliencyScore* ← *Mean (ComputeSaliencyMap(k))*20:      Compute *nccBlank* ← *ComputeNCC (k, ${template}_{blank}$)*21:      Compute *nccLogo* ← *ComputeNCC (k, ${template}_{logo}$)*22:      **if** *duration* < ${\Delta t}_{short}$ **then**23:            Remove *k* from *K*24:      **else if** *nccBlank* > *Threshold_Blank* **or** *nccLogo* > *Threshold_Logo* **then**25:            Remove *k* from *K*26:      **else if** *personArea* > *AreaThreshold* **and not** *HasContext(k)* **then**27:            Remove *k* from *K*28:      **else if** *ssim* > *SSIMThresholdHigh* **and** *duration* > *DurationThreshold* **then**29:            Remove *k* from *K*30:      **else if** *ssim* > *SSIMThresholdModerate* **and** *saliencyScore* < *SaliencyThresholdLow* **then**31:            Add *k* to *K’*32:      **end if**33:**end for**34:return *K, K’*

After TIB identifies pedagogically significant keyframes, the Spatial–Semantic Decoder (SSD) transforms each frame into a structured instructional representation. SSD operates in three steps: (1) an open-world proposal module detects potential educational components, (2) a segmentation module generates pixel-level masks for precise boundaries, and (3) verified region labels are used to construct the Educational Scene Graph as described in Algorithm 2. To ensure consistent and reliable annotations across large volumes of keyframes, LEARNet employs the Relational Consistency Verification Network (RCVN), which validates relationships between detected instructional elements such as figure → has → title and filters out predictions that are structurally or pedagogically inconsistent. By enforcing coherence across components, RCVN stabilizes SSD outputs and enables large-scale, low-supervision label propagation, ensuring high annotation quality throughout the EVUD-2M dataset.

This design enables accurate annotation with minimal supervision, requiring only ~200 reference frames, and supports large-scale propagation across the dataset. Through this process, we generated EVUD-2M, comprising over 949,000 keyframes with multi-level labels, including region masks and relational scene graphs. These annotations provide a strong foundation for advancing educational video understanding.

**Algorithm 2:** Spatial-Semantic Decoder (SSD)
1:**Input:** Test keyframe $I_{t}$, Reference set $R=\left({region\;}_{1},{label\;}_{1}\right),\ldots ,\left({region\;}_{k},{label\;}_{k}\right)$, Detection threshold $\tau_{detection}$, Similarity threshold *θ*2:**Output:** Educational scene graph *G*3:**// Level 1:** Open-World Object Proposal Model4:

$E_{t}\leftarrow EncodeImage\left(I_{t}\right)$

5:// Initialize reference embed6:dings7:**for each** $\left({region}_{r},{label\;}_{r}\right)\in R$ **do**8:      $E_{r}\leftarrow EncodeImage\left({region}_{r}\right)$9:      Store $\left(E_{r},{label\;}_{r}\right)\in$ in reference database $D_{ref}$10:
**end for**
11:Initialize *B* ← *∅* // Set of detected bounding boxes12:**for each** *patch embedding $v_{j}^{t}$* **do**13:    **for each** $\left(E_{r},{label\;}_{r}\right)\in D_{ref}$ **do**14:            Compute *similarity* ← $\frac{\left(v_{j}^{t}\cdot E_{r}\right)}{∥v_{j}^{t}∥∥E_{r}∥}$15:            **if** *similarity* > *$\tau_{detection}$* **then**16:              $bbox\leftarrow \;Localize\;region\;{(v}_{j}^{t})$17:            B←B∪bbox18:              **break**19:            **end if**20:
**end for**
21:
**end for**
22:**// Step 2:** Pixel-Accurate Mask Refiner23:Initialize *M* ← *∅*24:
**for each do**
25:      $mask\leftarrow RefineSegmentation\left(I_{t},bbox\right)$26:        ${refined}_{bbox}\leftarrow BoundingBoxFromMask\left(mask\right)$27:        $M\leftarrow M\cup {refined}_{bbox},mask$28:
**end for**
29:**// Step 3:** Entropy reduction, verification, Semantic graph formation, and annotation30:Initialize *G* ← *ConstructSceneGraph(M)* using Verified region labels31:**for each** component c∈G **do**32:          ${candidate}_{region}\leftarrow ExtractRegion\left(I_{t},c.bbox\right)$33:          ${embedding}_{candidate}\leftarrow Embed\left({candidate}_{region}\right)$34:          ${best}_{similarity}\leftarrow -\infty$35:         $\;{best}_{label}\leftarrow UNDEFINED$36:      **for each** $\left(E_{r},{label\;}_{r}\right)\in D_{ref}$ **do**37:                    $similarity\leftarrow Similarity\left({embedding}_{candidate},E_{r}\right)$38:                **if** $similarity>{best}_{similarity}$ **then**39:                      ${best}_{similarity}\leftarrow \;similarity$40:                     $\;{best}_{label}\leftarrow {label\;}_{r}$41:                **end if**42:      **end for**43:      **if** ${best}_{similarity}>\theta$ **then**44:              $c.label\leftarrow {best}_{label}$45:      **else**46:              $\;c.label\leftarrow InitialLabel\left({candidate}_{region}\right)$47:      **end if**48:
**end for**
49:**return** *G*


### 4.2. Temporal Information Bottleneck (TIB)

The TIB introduces a novel paradigm for educational video segmentation by reframing keyframe extraction as a problem of identifying frames with high information content. Unlike conventional video analysis methods that rely on a single-modality or visual cue, TIB follows a structured process built on two components:

Multi-Modal Information Analysis that computes a unified Pedagogical Information Score (PIS) by combining structural consistency cues with content-aware saliency to capture both major scene transitions and subtle instructional changes; and Informative Frame Selection and Redundancy Pruning which applies domain-specific rules to retain pedagogically meaningful frames while removing repetitive or visually redundant ones.

A core concept guiding TIB is the Instructional Steady-State (ISS), defined as intervals where teaching content evolves gradually and maintains low combined visual–semantic entropy. Formally, ISS is characterized by a unified entropy measure as defined in Equation (1):

(1) $$H_{inst}\left(t\right)=\propto H_{\upsilon }\left(t\right)+\left(1-\alpha \right)H_{s}\left(t\right)$$

$H_{\upsilon }\left(t\right)$ is the visual entropy of frame t, $H_{s}\left(t\right)$ semantic entropy over educational content categories (tables, diagrams, equations, text, etc.) and $\propto \in \left[0,1\right]$ balances visual and semantic contributions. As defined in Equation (2) interval $\left[t_{start},t_{end}\right]$ is considered an ISS segment if:

(2) $$H_{inst}\left(t\right)<\tau \forall t\in \left[t_{start},t_{end}\right],t_{end}-t_{start}\geq ∆t_{min}$$

Frames within ISS intervals are selectively retained by TIB as instructionally stable, while frames outside these intervals are suppressed, effectively filtering the video stream based on pedagogical significance. ISS therefore provides the criterion that enables TIB to align keyframe selection with meaningful instructional change.

Grounded in Shannon’s Information Theory, TIB seeks to minimize temporal redundancy in the video stream. The raw uncertainty of the frame sequence is quantified by entropy, as in Equation (3)

(3) $$H\left(X\right)=-\sum_{i=1}^{n}P\left(x_{i}\right)\log_{2}P\left(x_{i}\right)$$

In the context of instructional video, X represents the random variable over the frame sequence, and high $H\left(X\right)$ signifies the high informational redundancy—or visual noise—of the raw video stream. The degree of entropy reduction is quantitatively measured by the Entropy Reduction Rate $\left(R_{ER}\right)$ as in Equation (29), reflecting how effectively semantically irrelevant redundant frames are filtered out. Through this formulation, TIB filters the video into a compact set of instructionally rich frames, bridging the gap between low-level visual features and high-level educational semantics.

Temporal Preprocessing: The process begins with the extraction of individual frames from the input video. A critical preparatory step is the computation of each frame’s on-screen duration, $T\left(fᵢ\right)$, which is derived either from precise timestamps $\left(T\left(fᵢ\right)=fᵢ.timestamp-f{ᵢ}^{-1}.timestamp\right)$, or estimated from the frame rate $\left(T\left(fᵢ\right)=1/fps\right)$. This temporal information is foundational for subsequent domain-informed filtering.

**Figure 3:**
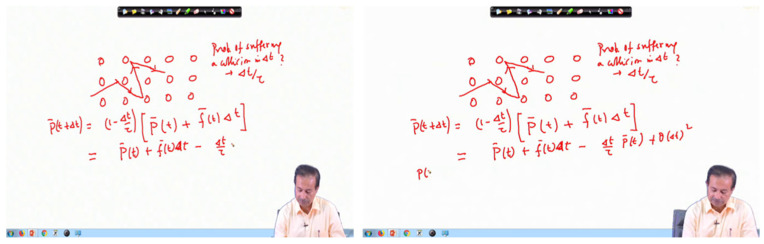
Limitation of I-frames in Educational Contexts: I-frames detect encoder-level scene changes but are insensitive to the subtle, pedagogically critical evolution of content within a lecture, demonstrating the need for a semantic-aware model.

Traditional video compression relies on three frame types—I-frames, P-frames, and B-frames—where I-frames (Intra-coded frames) are typically treated as natural keyframes due to their independent encoding and scene-representative properties. However, in educational videos, I-frames fail to capture incremental pedagogical changes, such as a lecturer gradually adding content to a slide. For instance, as illustrated in Figure 3, a lecturer incrementally adding content to a slide may not trigger an I-frame insertion, yet these evolving elements carry high instructional value. Conversely, an I-frame generated due to minor camera adjustments or lighting changes captures no meaningful educational transition.

This disconnect creates a critical methodological gap: while I-frames serve technical purposes in video compression, they fail to align with educational semantics. Our temporal coherence model addresses this by moving beyond compression artifacts to instead detect instructionally meaningful transitions, those moments where pedagogical content genuinely evolves, regardless of how they are encoded. This paradigm shifts from syntactic to semantic frame selection forms the cornerstone of our approach to educational video understanding.

Multi-modal information scoring: To detect meaningful transitions, the TIB calculates a Pedagogical Information Score that fuses multiple complementary visual cues to detect instructionally meaningful transitions, moving beyond mere pixel-level dissimilarity. This process, detailed in Algorithm 1 (Lines 2–10), moves beyond single-metric analysis to capture both structural changes and semantic content evolution. This score bridges the “instructional semantic gap” by fusing:

1. Structural dissimilarity: The model first quantifies macro-level scene changes through pixel-wise dissimilarity analysis between consecutive frames. This captures significant visual transitions such as slide changes or scene shifts, serving as the foundational signal for major content boundaries. This represents a coarse measure of signal-level change. The structural consistency between two frames is calculated using MSE as given in Equation (4):

(4) $$MSE\left(F_{i},F_{i-1}\right)=\frac{1}{N}\sum_{k=1}^{N}{\left(I_{i}\left(k\right)-I_{i-1}\left(k\right)\right)}^{2}$$

$I_{i}\left(k\right)$ is the intensity of pixel k in frame $F_{i}$ and N is the total number of pixels in the frame.

2. Content Saliency Analysis: To address the limitation of structural metrics in detecting subtle pedagogical changes, we incorporate content-aware saliency mapping. Identifies regions of visual importance within each frame, crucial for capturing incremental content additions (e.g., handwritten equations) that may not trigger significant pixel-level changes but constitute new information. The saliency score for a pixel p in frame F can be computed as in Equation (5):

(5) $$S\left(p\right)=\;\left|I\left(p\right)-\frac{1}{R}\sum_{r\in N\left(p\right)}I\left(r\right)\right|$$

$S\left(p\right)$ is the saliency score, which measures the contrast of pixel p relative to its neighborhood. is the intensity of the pixel $I\left(p\right)$. is the size of the neighborhood $N\left(p\right)$ around pixel $p_{1}$. $\sum_{r\in N\left(p\right)}I\left(r\right)$ is the average intensity of the neighboring pixels in the region around pixel $p_{1}$

3. Pedagogical Information Score (PIS): The structural and saliency components are integrated through an adaptive weighting scheme to compute the unified Pedagogical Information Score (Algorithm 1, Line 6). This is used in conjunction with the ISS principle to identify instructionally stable frames. The weight parameter α = 0.67, optimized empirically, balances the contribution of broad structural changes against focused content saliency, ensuring robust performance across diverse educational video formats. Unlike conventional saliency fusion, Equation (6) derives its adaptive weight α from the entropy of frame-level instructional cues, aligning selection with the information bottleneck principle.

(6) $$PedagogicalScore\left(F_{i}\right)=\alpha \cdot MSE\left(F_{i},F_{i-1}\right)+\left(1-\alpha \right)\cdot mean\left(SaliencyMap\;\left(F_{i}\right)\right)$$

α is the threshold weight assigned to the MSE metric $\left(\alpha =0.67\right)$ that is empirically chosen to prioritize structural change while retaining saliency information. Frames with higher $AWS\left(F\right)$ values are considered more significant for keyframe selection, as they have both higher visual differences and important content.

4. Instructional Transition Identification: The temporal sequence of Pedagogical Scores undergoes Density Peak Clustering (DPC) (Algorithm 1, Line 9) to identify the most significant and non-redundant frames. This clustering approach operates on the principle that instructionally important frames form natural density peaks in the feature space, with cluster centroids representing optimal keyframe candidates.

The DPC process effectively groups frames that are temporally and visually similar, representing periods of high-entropy redundancy (or low instructional change). The critical step of the Temporal Information Bottleneck (TIB) module is the selection of a single, optimal keyframe from each cluster to pass through to the next stage.

Crucially, we define the optimal keyframe as the one possessing the Maximum Instructional Content Value $\left(c_{value}\right)$ within its cluster. To identify this frame, we calculate a unified score (the Pedagogical Score) for every frame, ensuring the frame chosen is the most visually clear and semantically rich realization of the cluster’s content. The local density of a frame is defined as in Equation (7):

(7) $$\rho_{i}=\;\sum_{j\neq i}exp\left(-\frac{(PedagogicalScore{\left(F_{i}\right)-PedagogicalScore\left(F_{j}\right))}^{2}}{2\sigma^{2}}\right)$$

σ is a parameter controlling the smoothness of the similarity decay. The cut-off distance $\delta_{i},$ for each frame is defined as in Equation (8) by comparing the local density of frame i to that of its neighbors:

(8) $$\delta_{i}=\underset{j:\rho_{j}>\rho_{i}}{\min}\left(PedagogicalScore\left(F_{j}\right)-(PedagogicalScore\left(F_{i}\right)\right)$$

This multi-modal fusion strategy enables the model to detect both overt scene transitions and subtle content evolutions, effectively bridging the gap between low-level visual features and high-level pedagogical significance that characterizes educational video content.

Domain Informed Pruning: After candidate frames are selected, a refinement stage removes non-informative or repetitive visuals through domain-informed pruning (Algorithm 1, Lines 11–20). The temporal sequence of Information Scores undergoes clustering to identify the most significant and non-redundant frames. Cluster centroids represent optimal keyframe candidates. This candidate set is then refined through domain-informed pruning, which applies educational context-aware filters to eliminate semantically redundant or pedagogically irrelevant frames, ensuring the final keyframe set is both information-dense and pedagogically valuable. The pruning rules include:

1. Temporal Significance Filters: Leveraging frame duration metadata, we prioritize frames with substantial exposition time while discarding transient content:
  - Extended duration Frames: Frames with extended screen time (Equation (9)) are prioritized as they often contain stable, pedagogically rich content.
    (9) $$T\left(f_{i}\right)>{\Delta t}_{long}\Rightarrow fi\in K$$
  - Brief segments: Frames with very brief screen time or short associated transcription length are considered transient and are discarded as in Equation (10).
    (10) $$T\left(f_{i}\right)<{\Delta t}_{short}\vee L_{transcript}\left(fi\right)<\theta_{transcript}\Rightarrow Discard\;f_{i}$$
2. Content Quality validation: We eliminate non-educational elements through template matching.
  - Blank Page and Logo Detection: Frames matching pre-defined templates for blank slides or institutional logos are detected using Normalized Cross-Correlation (NCC) and discarded to avoid processing non-content frames as in Equation (11).
    (11) $$NCC\left(f_{i},T_{blank}\right)>\theta_{blank}\vee NCC\left(f_{i},T_{logo}\right)>\theta_{logo}\Rightarrow Discard\;f_{i}$$
    NCC for images A and B is computed as in Equation (12):
    (12) $$NCC\left(A,B\right)=\frac{\sum_{i}\left(A_{i}-\bar{A}\right)\left(B_{i}-\bar{B}\right)}{\sqrt{\sum_{i}{\left(A_{i}-\bar{A}\right)}^{2}\sum_{i}{\left(B_{i}-\bar{B}\right)}^{2}}}$$
  - Instructional Context Verification: As mentioned in Equation (13), frames dominated by a human speaker without accompanying educational content (e.g., slides, text) are removed. A frame is discarded if the area of detected persons exceeds a threshold and a context-checking function (e.g., via object detection for slides/text) returns false.
    (13) $${Area}_{person}\left(f_{i}\right)>A_{threshold}\wedge \neg HasContext\left(f_{i}\right)\Rightarrow Discard\;f_{i}$$
3. Temporal Coherence Optimization: This step leverages the Structural Similarity Index Measure (SSIM) maintain semantic diversity while reducing redundancy.
  - High-similarity consecutive frames: Frames highly like their immediate predecessors are considered redundant and pruned as in Equation (14).
    (14) $$SSIM\left(f_{i},f_{i-1}\right)>\theta_{high}\Rightarrow Discard\;f_{i}$$
  - Moderate-Significance Bucketing: Frames that are moderately like previous keyframes and possess low visual saliency are deemed of secondary importance. These are not discarded but are stored in a separate set $K^{\prime }$ as in Equation (15) for potential use in applications requiring higher temporal density.
    (15) $$SSIM\left(f_{i},f_{prev}\right)>\theta_{moderate}\wedge Saliency\left(f_{i}\right)<\theta_{low}\Rightarrow f_{i}\in K^{\prime }$$

Concurrently, we optimized redundancy removal thresholds $\theta_{high}$ across [0.3, 0.5, 0.7, 0.9], and duration filtering parameters ${(\Delta t}_{long},\;{\Delta t}_{short})$ derived from educational content patterns. The optimal configuration ($\theta_{high\;}$ = 0.7, and $\;{\Delta t}_{long\;}$= 3.0 s) demonstrates that structural consistency contributes approximately twice as significantly as content saliency in identifying pedagogically meaningful transitions—aligning with the importance of slide changes and major content shifts in educational discourse. This domain-aware optimization ensures that the final keyframe set is compact yet conceptually rich, balancing computational efficiency with the preservation of essential educational details.

#### Parameter Optimization and Sensitivity Analysis

To ensure the reproducibility and robustness of the Temporal Information Bottleneck (TIB) module, a critical preliminary step involved the empirical determination of its core hyperparameter: the Pedagogical Information Score (PIS) weighting factor, α. This parameter determines the balance between structural consistency and content saliency within TIB and therefore shapes how instructional transitions are detected. A grid search over α∈{0.5,0.6,0.67,0.7,0.8} on the EVUD-2M training split identified α = 0.67 as the optimal configuration, providing the strongest keyframe-selection performance. Redundancy-filtering thresholds were tuned in a similar data-driven manner based on temporal patterns observed in instructional videos, and the selected values were used consistently throughout all experiments. A detailed evaluation of the α-sweep and its joint effect on keyframe selection and DPC behavior is presented in the Results and Analysis section.

### 4.3. Spatial Semantic Decoder (SSD)

**Figure 4:**
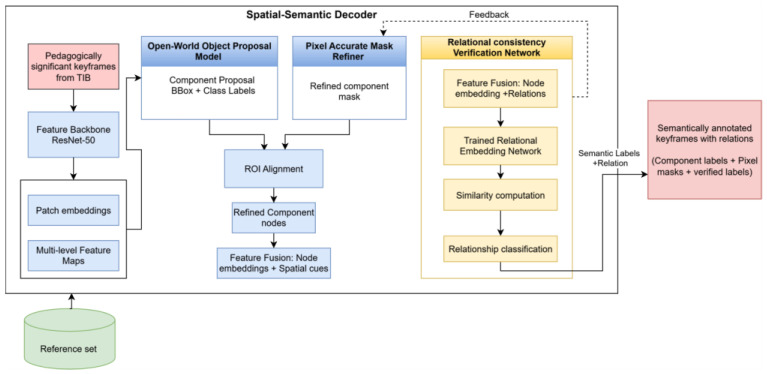
Spatial-Semantic Decoder (SSD) Architecture of the Spatial–Semantic Decoder (SSD) in LEARNet, illustrating the three-stage process of open-world detection, pixel-accurate mask refinement, and relational consistency verification to produce semantically structured educational annotations.

The Spatial-Semantic Decoder (SSD) forms the second stage of the LEARNet framework. Its role is to take the pedagogically significant keyframes produced by the TIB module and expand them into a complete, structured High-Fidelity Semantic Graph, as shown in Figure 4. Since the Temporal Information Bottleneck (TIB) standardizes all selected keyframes to an input resolution of 224 × 224, the SSD also receives its inputs at this same size, ensuring consistent processing across all subsequent stages. The SSD operates through three integrated modules: open-world component proposal, pixel-accurate component isolation, and the Relational Consistency Verification Network (RCVN). The name “Spatial-Semantic Decoder” reflects its coordinated functionality: it acts as a decoder by expanding compressed keyframe representations into full scene graphs; it is spatial in its emphasis on geometric reasoning and pixel-level precision; and it is semantic because it assigns pedagogical labels and verifies logical relations through the RCVN.

The Relational Consistency Verification Network (RCVN) forms the core innovation of the SSD, transitioning from independently detected regions to semantically verified components that are subsequently assembled into a structured, pedagogically meaningful scene graph. It verifies semantic label consistency by computing similarity-based relational cues, builds the Educational Element Relationship Graph, and then annotates its components using the validated labels. While the first two stages identify candidate regions and refine spatial boundaries, the RCVN evaluates semantic and relational consistency among educational elements—an ability absent in conventional detection pipelines. Once semantic consistency is confirmed, the scene-graph module uses these verified relationships to produce a structured graph in which nodes represent educational components and edges encode meaningful instructional relations.

This decoder operates through three integrated levels:

Open-World Object Proposal Model: The first level addresses the challenge of diverse educational content through open-world detection by generating object-level hypotheses using entropy-driven attention maps to capture pedagogically salient regions. We employ a vision-language model as a domain-agnostic proposal generator, enabling identification of both common and novel educational elements without class-specific retraining.

Given a keyframe image $I_{t}\in R^{H\times W\times 3}$ and a reference image $I_{r}\in R^{H\times W\times 3}$, as in Equation (16), Equation (17) encodes both into respective embedding spaces using a shared vision-language encoder ε.

(16) $$E_{t}=\varepsilon \left(I_{t}\right)\;\left(Patch-level\;embeddings\;of\;the\;test\;image\right)$$

(17) $$E_{r}=\varepsilon \left(I_{r}\right)\left(Globalembeddingofthereferenceimage\right)$$

The goal is to localize regions $R_{j}\subset \;I_{t}$ in the test image $I_{t}$ that semantically correspond to $I_{r}$. Semantic similarity between the patch embeddings $v_{j}^{t}\;$ of $I_{t}$ and the global embedding of $I_{r}$, is computed as in Equation (18).

(18) $$s_{j}=\frac{\left(v_{j}^{t}\cdot E_{r}\right)}{∥v_{j}^{t}∥∥E_{r}∥}$$

$v_{j}^{t}\in$ $E_{t}$ is the embedding of the j−th rejoin in the test image, $E_{r}$ is the embedding of the reference image. $s_{j}$ is the similarity score for rejoin j. Bounding boxes $b_{j}$ are selected for regions where this semantic similarity $s_{j}$ exceeds a predefined detection threshold $\tau_{detection}$ as in Equation (19).

(19) $$b_{j}\leftarrow LocalizeRegion\left(v_{j}^{t}\right)\;where\;s_{j}>\tau_{detection}$$

The highest-scoring regions are selected as candidate detections. This allows the system to generalize to novel object categories (e.g., diagrams, equations, slide figures) with minimal supervision. These highest-scoring regions, represented by their bounding boxes $b_{j}$, are considered candidate detections. The misalignment can be measured using the Intersection over Union (IoU) metric as in Equation (20).

(20) $$IoU\left(b_{j},b_{j}^{*}\right)=\frac{Area\left(b_{j}\cap b_{j}^{*}\right)}{Area\left(b_{j}\cup b_{j}^{*}\right)}$$

Bounding boxes are considered precise if $IoU\left(b_{j},b_{j}^{*}\right)<\tau$, where τ is a chosen threshold. This stage is critical for educational elements with complex boundaries such as handwritten formulas, diagrams, or annotated illustrations, where spatial precision directly impacts downstream tasks like OCR and layout analysis.

Pixel-accurate mask refiner (PAMR): The second level refines coarse proposals into precise segmentation masks using a foundational segmentation model. It refines spatial boundaries and suppresses redundant background information using entropy-adaptive masking. The innovation lies in the intelligent orchestration—using bounding box proposals as precise prompts to guide segmentation exclusively onto pedagogically relevant regions.

Formally, for each test keyframe $I_{t}\in R^{H\times W\times 3}$, and a bounding box proposal $b_{j}$, a binary segmentation mask as in Equation (21).

(21) $$M_{j}=f_{sam}\left(I_{t},b_{j}\right)$$

$M_{j}\in {\left\{0,1\right\}}^{H\times W},\;\;M_{j}\left[\upsilon ,\nu \right]=1\;$ implies pixel $\left(\upsilon ,\nu \right)$ belongs **to** the object in **region** $b_{j}$. The refined bounding box $\tilde{b_{j}}$, which tightly encloses the segmented region, is computed as in Equation (22).

(22) $$\tilde{b_{j}}=BBox\left(M_{j}\right)=\underset{\mathrm{rect}}{\min}\left(\left(\upsilon ,\nu \right)|M_{j}\left[\upsilon ,\nu \right]=1\right)$$

This refinement is particularly critical for complex educational visual structures, such as diagrams, mathematical expressions, or handwritten annotations, where precise boundaries directly impact the effectiveness of downstream tasks like optical character recognition (OCR), layout analysis, and diagram parsing. The refined regions are then forwarded to the RCVN for semantic and relational consistency evaluation.

Relational Consistency Verification Network (RCVN): The proposed RCVN is a dual-stream verification model designed to assess pedagogical consistency between educational content regions. The Relational Consistency Verification Network (RCVN) operates as the final verification stage in the annotation pipeline. Its role is to evaluate whether a region extracted from the educational content displays semantic consistency with any region in the curated reference database.

**Figure 5:**
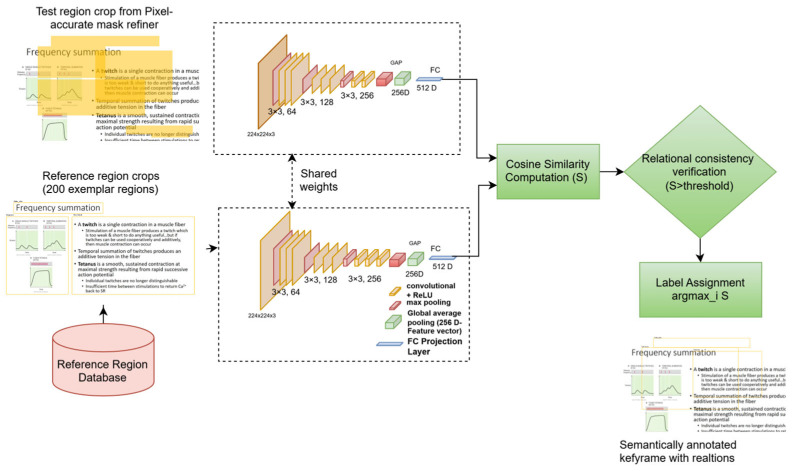
Overview of the Relational Consistency Verification Network. Refined region crops and reference exemplars are transformed into 512-D normalized embeddings and compared using cosine similarity. A similarity threshold determines relational consistency, and the final label is selected via maximum validated similarity.

The Relational Consistency Verification Network (RCVN), as depicted in Figure 5, determines whether a detected region in the test keyframe corresponds semantically to any exemplar region stored in the reference database. Each test crop produced by the pixel-accurate mask refiner is compared against a curated set of 200 reference regions representing common educational components such as diagrams, equations, slide titles, and text blocks.

Both the test regions and the reference regions are resized to 224 × 224 × 3 and passed through a shared-weight feature-extraction backbone to ensure uniform representation. This backbone consists of three convolutional layers with 3 × 3 kernels and filter dimensions of 64, 128, and 256, respectively, each followed by max pooling and ReLU activation. The resulting feature maps are aggregated using Global Average Pooling to produce a compact 256-dimensional descriptor, which is then projected into a 512-dimensional semantic embedding through a fully connected projection layer. Cosine similarity is computed between the embedding of the test region and each reference embedding, producing a similarity score, *S*. A region is considered semantically consistent when its similarity exceeds a predefined threshold. The final label is assigned by selecting the reference exemplar that yields the maximum validated similarity score, resulting in a semantically annotated keyframe enriched with relational information.

Given a region $R_{t}^{i}$ from test image and a reference region $R_{r}^{j}$, the Relational Consistency Verification network compares their embeddings via a similarity function S, typically based on cosine similarity, where the L2 norm is used for normalization before computing the cosine score as in Equation (23).

(23) $$S\left(R_{r}^{i},R_{t}^{j}\right)=cos\left(f_{cnn}\left(R_{r}^{i}\right),f_{cnn}\left(R_{t}^{j}\right)\right)=\frac{f_{cnn}\left(R_{r}^{i}\right)\cdot f_{cnn}\left(R_{t}^{j}\right)}{{∥f}_{cnn}\left(R_{r}^{i}\right)∥\cdot ∥f_{cnn}\left(R_{t}^{j}\right)∥}$$

If $S\left(R_{r}^{i},R_{t}^{i}\right)>\theta ,$ where θ is a similarity threshold, the region $R_{t}^{i}$ is confirmed as a match for class $c_{i}$, inherited from $R_{r}^{i}$. This enables label transfer and spatial refinement even in the absence of direct supervision for $R_{t}^{i}$.

The final refined label for $R_{t}^{j}$ is given as in Equation (24).

(24) $${\hat{c}}_{j}=arg\;\underset{i}{\max}\left(R_{r}^{i},R_{t}^{j}\right)$$

*Training and Hyperparameter Optimization*: RCVN is trained using a Triplet Loss objective formulated to maximize relational separability. For each training step, the network processes as in Table 5: an anchor region, a semantically consistent reference region (positive), and a semantically inconsistent region (negative). The loss function enforces a margin of 0.2 between positive and negative similarity distances, encouraging compact relational clusters while maintaining strong inter-class separation. We employ AdamW as the optimizer with a learning rate of 1 × 10^−3^, weight decay of 0.05, and a batch size of 32, training for 50 epochs. To enhance fine-grained discrimination, particularly for visually similar but semantically different educational content, hard negative mining is incorporated. This strategy selects the most challenging negative samples—those with high accidental similarity—to refine the embedding space. Throughout training, the 200-region reference database provides consistent exemplars that anchor the relational embedding manifold.

*Domain-specific validation protocol*: RCVN operates on TIB-selected keyframes (~1.5% of video frames), ensuring computational efficiency while maintaining pedagogical relevance. The framework incorporates a domain-specific validation protocol, enabling accurate annotation of 2M frames using only 200 reference frames (~10,000:1 ratio). This protocol addresses:

- Pedagogical ambiguity resolution (distinguishing diagrams, equations, and complex illustrations)
- Structural diversity management (consistent labeling across slides, blackboards, and digital writing)
- Temporal consistency maintenance across evolving lecture segments

Domain experts manually annotated a stratified reference set using a pedagogically grounded taxonomy, achieving high inter-annotator agreement (κ = 0.92). This educationally curated foundation enables reliable semi-automatic propagation while maintaining quality through curriculum-aware validation checks against 95% accuracy thresholds.

Our education-optimized validation protocol ensures spatially precise and semantically consistent annotations at scale, overcoming both the scalability limitations of traditional manual annotation and the domain-specific challenges of educational content understanding.

#### 4.3.1. Model Optimization

All model parameters were empirically optimized on the EVUD-2M training split to ensure stable and reproducible performance across the LEARNet pipeline. For the Temporal Information Bottleneck (TIB), the Pedagogical Information Score (PIS) weighting factor *α* was tuned through grid search over {0.5,0.6,0.67,0.7,0.8}, with α = 0.67 achieving the best balance between structural consistency and content saliency. The same sweep also determined the induced Density Peak Clustering (DPC) cut-off behavior, and the selected value was used consistently in all experiments. Redundancy-pruning thresholds and duration constraints were tuned in a similar data-driven manner based on instructional temporal patterns.

For the Spatial–Semantic Decoder (SSD), the open-world detection threshold $\tau_{detection}$ for Open World Object Proposal Model was optimized over [0.1, 0.3, 0.5, 0.7, 0.9], selecting $\tau_{detection}$ = 0.5 for optimal precision-recall balance in educational element detection. The SSD was trained using the AdamW optimizer (learning rate $2\times 10^{-4}$), a cosine annealing scheduler with 5-epoch warmup, a batch size of 16, and 50 training epochs. The SSD loss function combined object-proposal contrastive loss and segmentation refinement loss to stabilize spatial predictions. For the proposed Relational Consistency Verification Network (RCVN), hyperparameters such as embedding dimension, learning rate, batch size, and the Triplet Loss margin were tuned to ensure stable relational discrimination. Hard negative mining was adopted to refine the embedding space, and all final settings were fixed for consistency across training and evaluation.

Finally, an end-to-end fine-tuning stage (with TIB frozen) was conducted to harmonize SSD and RCVN representations. This phase used a learning rate of $2\times 10^{-5}\;for\;$20 epochs, enabling joint refinement of spatial and semantic components while preserving the entropy-aware temporal structure learned by TIB.

#### 4.3.2. Loss Function and Learning Strategy

The Hierarchical Educational Scene Parser in LEARNet integrates frozen foundation models with a lightweight, trainable verification module. Rather than employing end-to-end training, LEARNet leverages fixed vision–language and segmentation backbones [28,69] to preserve their zero-shot generalization capacity while allowing the relational layer to adapt specifically to educational content. These backbones provide stable region proposals and pixel-accurate masks, forming a reliable basis for higher-level semantic reasoning.

Relational Consistency Verification Network- Loss Function: The trainable component of the system is the RCVN, which learns a coherence-driven embedding space using Triplet Loss. The objective function is expressed in Equation (25).

(25) $$L_{triplet}=\frac{1}{N}\sum_{i-1}^{N}max\left(0,{∥f\left(x_{a}^{i}\right)-f\left(x_{p}^{i}\right)∥}_{2}^{2}-{∥f\left(x_{a}^{i}\right)-f\left(x_{n}^{i}\right)∥}_{2}^{2}+m\right)$$

We represent $x_{a}^{i}$ (anchor) and $x_{p}^{i}$ (positive) as patches from the same semantic class, $x_{n}^{i}$ (negative) is a patch from a different class, $f\left(x\right)$ is the embedding generated by the Relational Verification network, m is a margin hyperparameter, and N is the number of triplets. This optimization ensures that pedagogically similar regions are embedded in proximity, while educationally distinct elements are separated in the feature space. The margin parameter m controls the separation degree, enforcing semantic boundaries that align with educational categorization.

Loss Formulation for Relational Coherence: Instead of conventional classification losses (e.g., binary cross-entropy), RCVN uses Triplet Loss that allows the model to learn relationships in a relative, context-aware manner. $L_{triplet}$ allows the SSD to learn a coherence embedding space where the distance between an Anchor feature (e.g., a Diagram) and a Positive feature (e.g., its explaining Text box) is enforced to be less than the distance to a Negative feature (e.g., a random, unrelated Professor in the corner), separated by a fixed margin m. This relational learning strategy helps the model generalize to unseen instructional layouts, ensuring that semantic associations remain consistent even when visual structures vary widely. By constructing a coherence-driven embedding space rather than relying on fixed categorical boundaries, the SSD becomes more resilient to noise and avoids propagating weak or non-instructional relationships that typically degrade the quality of conventional scene graph models.

#### 4.3.3. Computational Efficiency and Trade-Offs

Although the Spatial–Semantic Decoder (SSD) performs fine-grained segmentation and relational parsing, the overall system remains efficient due to the entropy-aware Temporal Information Bottleneck (TIB). By discarding more than 70% of frames before spatial processing, TIB ensures that SSD is applied only to instructionally meaningful keyframes. This design choice offers a favorable trade-off: the computational cost of pixel-level parsing is justified by the substantial reduction in temporal redundancy.

### 4.4. Integrated Framework and EVUD-2M Benchmark Construction

The integrated LEARNet pipeline enables scalable creation of the EVUD-2M benchmark. TIB first extracts approximately 949,000 pedagogically significant keyframes from 2 million raw frames. The SSD then propagates fine-grained region-level annotations from a small set of 200 reference frames, achieving a 10,000:1 annotation expansion ratio while maintaining semantic consistency through RCVN verification. The final output, the EVUD-2M benchmark, represents the first large-scale educational video resource that combines:

- Temporal pedagogical coherence through carefully selected keyframes
- Spatial semantic richness with region-level annotations for educational elements
- Structural diversity covering slides, diagrams, equations, and handwritten content

This benchmark establishes a foundational resource for developing and evaluating next-generation educational video understanding systems, addressing the critical gap in large-scale, fine-grained educational video data.

### 4.5. Implementation and Experimental Framework

LEARNet was implemented in Python 3.9 and PyTorch 2.1.0 as a unified system integrating TIB and the Hierarchical Scene Parser. The RCVN employs batch normalization after each convolutional layer for stable training across heterogeneous educational content. A key implementation innovation lies in the Relational Verification Network, which incorporates batch normalization following each convolutional layer to maintain stable learning dynamics across diverse educational content types. This architectural choice proved crucial for handling the varied visual characteristics of instructional materials from dense blackboard content to structured slide presentations within a single unified model. A 5-fold cross-validation protocol (80% train, 20% test) was used for all experiments. Models were trained with Adam (learning rate 0.001, batch size 32), and hard-negative mining was applied to refine relational discrimination. All experiments were conducted on dual NVIDIA RTX 4090 GPUs (48GB VRAM), and 24-core processing capability enabling efficient large-scale processing and relational reasoning.

## 5. Results and Analysis

This section presents a comprehensive evaluation designed to validate a core thesis: that a framework explicitly designed for the Instructional Steady-State principle significantly outperforms both conventional methods and general-purpose foundation models on educational video understanding. We demonstrate this through quantitative benchmarks, qualitative analysis, and rigorous ablation studies on the EVUD-2M benchmark. The dataset used contains only structured instructional formats (slides, static writing surfaces) and excludes dynamic classroom interactions outside the current scope.

### 5.1. Evaluation Framework and Metrics

This section evaluates the proposed LEARNet framework and validates its effectiveness in educational video understanding. The assessment focuses on two main goals: (1) selecting keyframes that capture meaningful instructional transitions, and (2) accurately identifying educational components such as text, diagrams, and handwritten content. Quantitative assessment utilized four standard metrics—precision, recall, F1-score, and mAP@50—with their formal mathematical representations as in Equations (26)–(28).

(26) $$Precison=\frac{TP}{TP+FP}$$

(27) $$Recall=\frac{TP}{TP+FN}$$

(28) $$F1-SCORE=2\cdot \frac{Precision\cdot Recall}{Precision+Recall}$$

TP represents true positives (correctly identified keyframes/objects), TN represents true negatives (correctly rejected non-keyframes/non-objects), FP represents false positives (incorrectly selected keyframes/objects), and FN represents false negatives (missed keyframes/objects).

The effectiveness of the TIB is quantified using the Entropy Reduction Rate $\left(R_{ER}\right)$, which measures the percentage of visually redundant frames ensuring that only content-rich keyframes proceed for semantic decoding (SSD). Maximizing this metric directly improves the system’s overall efficiency and learning focus. $R_{ER}$ is calculated as in Equation (29).

(29) $$R_{ER}=1-\frac{\left|KeyframesselectedbyTIB\right|}{\left|TotalVideoFrames\right|}$$

The mean Average Precision (mAP@50) was calculated by averaging the precision values across all recall levels for each object category, considering detections with an Intersection over Union (IoU) greater than 0.5 with ground truth annotations as true positives.

All experiments were conducted on the EVUD-2M benchmark using a standardized 70/15/15 train/validation/test split. Keyframe selection was evaluated frame-by-frame against manually annotated ground truth, while semantic detection performance was assessed across all educational element categories. Statistical significance was determined using paired *t*-tests (*p* < 0.05).

### 5.2. Comparative Baselines: Establishing the State of the Art

To contextualize our performance, LEARNet was compared against three major baseline categories: traditional keyframe methods, modern detection systems, and specialized educational frameworks.

#### 5.2.1. Keyframe Extraction Baselines

The VSUMM approach [70] employs color histogram differences for keyframe selection, making it effective for general video summarization but inadequate for educational content where significant semantic transitions occur with minimal color variation. Motion-Based Detection [18] utilizes optical flow to identify frames with significant movement, performing well on dynamic content but failing on educational videos characterized by static camera setups and pedagogically important static content. Multimodal CLIP + Optical Flow Saliency [22] combines multimodal embeddings with temporal cues, offering improved performance but requiring substantial computational resources (3.2 s per frame) while remaining sensitive to text density variations.

#### 5.2.2. Semantic Annotation Baselines

Faster R-CNN [71] provides robust closed-vocabulary detection but requires extensive category-specific training and cannot handle novel educational elements like handwritten equations or domain-specific diagrams. The Lecture Video Visual Objects (LVVO) dataset [72], while education-specific, relies on Faster R-CNN and achieves only 0.72 mAP@50, demonstrating the limitations of conventional detection approaches in educational contexts.

Modern Detection and Segmentation Models represent the current state-of-the-art but exhibit critical limitations: OWL-ViT produces coarse bounding boxes unsuitable for precise educational annotation; SAM offers excellent segmentation but lacks semantic labeling; GLIP depends heavily on textual prompts, limiting effectiveness for diagram-heavy content. OWL-ViT (Without Refinement): The standalone OWL-ViT model [51] performs open-world object detection using vision-language similarity. While capable of zero-shot detection, it produces coarse bounding boxes with poor spatial precision, particularly for thin or irregular educational elements. Without segmentation refinement, it achieves only 0.73 mAP@50 on educational content, highlighting the need for the cascaded refinement approach proposed in our CSAM framework. CLIP-ViP [73] is a CLIP-based video understanding pipeline that employs frame-text alignment for content segmentation. GPT-4V [74] utilizes prompt-based analysis of keyframes with educational context descriptions. VideoCLIP [75] enables temporal extension of CLIP for video–text matching.

#### 5.2.3. Specialized Educational Systems

LectureNet and EduNet provide basic keyframe extraction but lack region-level annotations and support for diverse educational formats. AI2D/ChartQA focus on diagram understanding but do not support video content or lecture-style presentations.

Collectively, these baselines establish a shared limitation in educational video understanding: traditional methods rely on low-level features unsuitable for educational semantics, while general-purpose models lack the fine-grained structure needed for educational understanding. All compared methods were trained and evaluated on the same EVUD-2M dataset to ensure fair comparison.

### 5.3. LEARNet Performance Analysis

#### 5.3.1. Optimization of Pedagogical Information Score (PIS)

**Figure 6:**
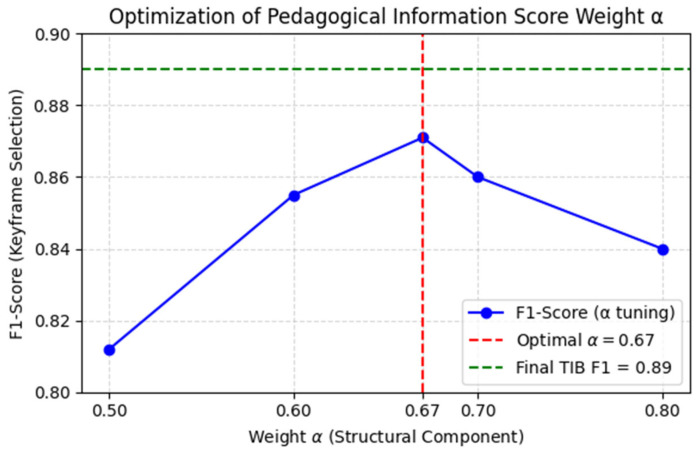
Optimization of Pedagogical Information Score Weight α—F1-Score for Keyframe Selection and Final TIB Performance.

The Pedagogical Information Score (PIS) is utilized within the TIB module to select instructionally stable keyframes by integrating structural change and saliency components. The weight α dictates the balance between detecting instantaneous structural changes (high MSE) and confirming the presence of pedagogically relevant content (high saliency). To empirically determine the optimal value of α, we conducted a tuning experiment evaluating keyframe selection performance across a range of plausible weights, specifically α∈{0.5,0.6,0.67,0.7,0.8}. The evaluation metric used was the F1-score against the human-labeled ground truth dataset. The results of this optimization process are summarized in Table 6 and visualized in Figure 6.

The results, summarized in Table 1 and visualized in Figure 6, show that α = 0.67 yields the highest F1-score (0.871). Lower values (0.5 or 0.6) select redundant frames due to insufficient emphasis on structural transitions, while higher values (0.7 or 0.8) discard saliency cues and reduce recall. The chosen value of α therefore provides the most effective balance between detecting meaningful pedagogical transitions and suppressing visually similar frames.

#### 5.3.2. Value Basis and Sensitivity of the DPC Cut-Off Parameter

The DPC cut-off distance (σ) controls the neighborhood size for density computation in the Instructional Transition Identification stage. Its selection directly affects which frames are considered density peaks, and thus the quality and number of extracted keyframes. To ensure σ reflects the intrinsic structure of the PIS distribution, we employ a percentile-based heuristic: σ is set to the 2nd percentile of all pairwise PIS distances, making it small enough to capture local variations but robust against noise. Because the PIS distribution depends on the weighting factor α, σ naturally varies with α, motivating a sensitivity analysis.

Table 7 reports the results for a representative lecture video: Flight Dynamics II (Stability), IIT Madras, Mod-01 Lec-01 (FPS = 25, Total Frames = 71,911, Duration = 2876 s). Although σ is computed per video, the trends observed here—higher α produces larger σ due to wider PIS distributions, lower α compresses σ—are consistent across the dataset. Presenting this single representative case clearly demonstrates the characteristic dependency of σ on α without redundancy, and highlights that at α = 0.67, σ achieves a moderate value that balances sensitivity and noise suppression. Although σ is computed per video, the qualitative trend is consistent across the dataset: higher α emphasizes structural saliency, widening the PIS distribution and producing larger σ values, while lower α compresses the PIS range and yields smaller σ. Since σ is derived from pairwise distances in the Pedagogical Information Score distribution, and α modulates this distribution, σ varies implicitly with α. Hence, α directly influences the density landscape used by DPC during transition detection. Presenting one representative example provides a clear and interpretable demonstration of this characteristic behavior without redundancy.

**Figure 7:**
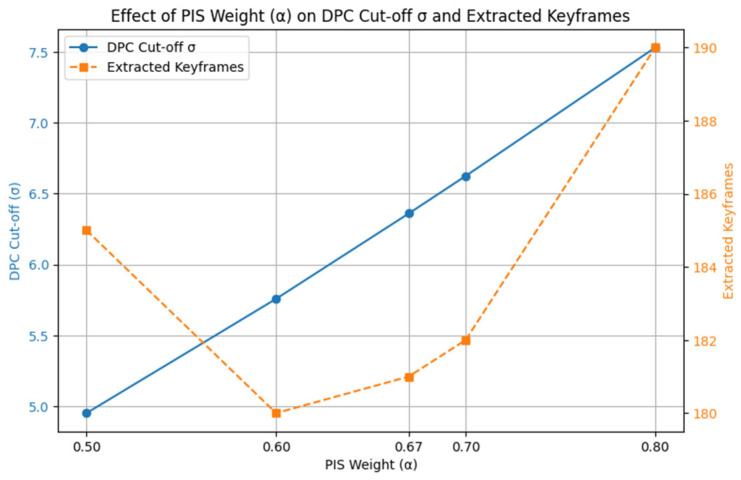
Sensitivity of the DPC cut-off parameter $\left(\sigma \right)\;$ to changes in PIS Weight (*α*).

Figure 7 illustrates the sensitivity of the DPC cut-off σ to changes in PIS Weight. While σ increases steadily with α, the number of extracted keyframes remains largely stable, confirming that α=0.67 achieves an optimal balance for robust instructional transition detection.

**Figure 8:**
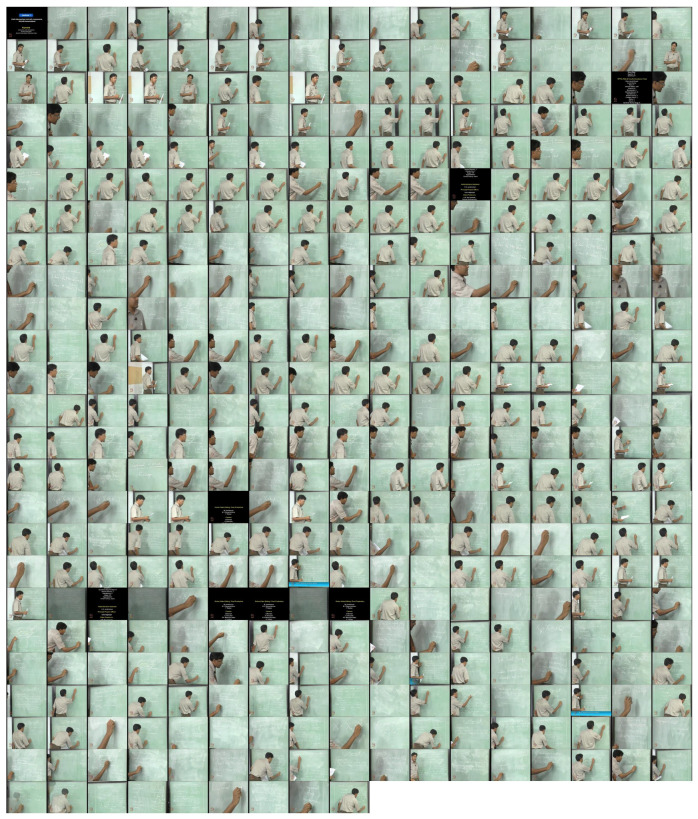
Keyframes from a representative instructional video used to validate the sensitivity of α and *σ*. Although the entire dataset was analyzed this example clearly illustrates how α = 0.67 achieves stable and balanced keyframe extraction across diverse instructional transitions.

Figure 8 illustrates keyframes extracted from a representative instructional video, characterized by frequent board writing and instructor movement. This video was selected to validate the sensitivity of the PIS Weight (α) and the DPC cut-off parameter $\left(\sigma \right)$ as it contains a variety of instructional transitions and structural changes, providing a clear visual demonstration of the effect of these parameters. While the entire dataset was used to compute optimal α and σ values, this example is presented for clarity, as it effectively highlights the balance achieved at α=0.67, where keyframe extraction is both stable and representative of typical instructional content.

#### 5.3.3. TIB Performance for Pedagogical Keyframe Extraction

The Temporal Information Bottleneck (TIB) demonstrates superior performance in identifying pedagogically significant frames, achieving an F1-score of 0.89 while reducing data volume by 70.2% (Table 8). This shows that TIB effectively distinguishes pedagogical transitions from visual noise, maintaining essential instructional information while minimizing redundancy.

Redundancy reduction analysis: The hierarchical filtering approach ensures that only pedagogically relevant frames are processed, reducing computational overhead while maintaining content integrity. The model’s efficiency stems from its hierarchical filtering approach (Table 4), which systematically eliminates non-informative content while preserving instructional value. This domain-aware design ensures computational resources focus exclusively on educationally rich frames.

**Figure 9:**
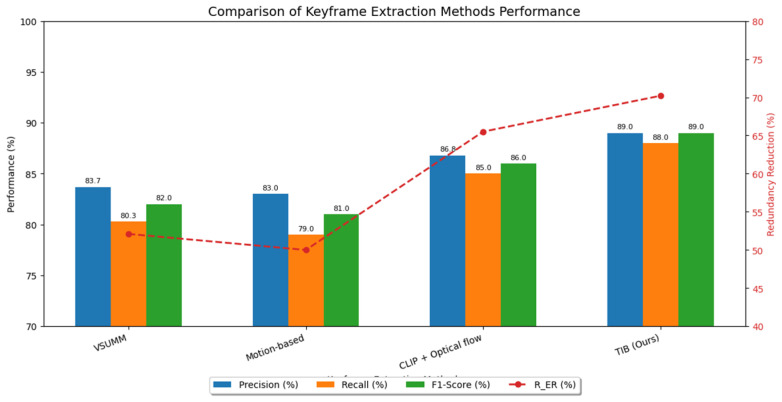
Comprehensive comparison of keyframe extraction performance across multiple evaluation dimensions.

Figure 9 provides a comprehensive visualization of keyframe extraction method comparisons across multiple performance dimensions. The results confirm that TIB not only identifies the most pedagogically relevant frames but also removes unnecessary visual redundancy. By focusing on entropy reduction rather than frame count, it preserves the instructional flow while cutting data volume by over 70%. Traditional baselines such as VSUMM or motion-based detection rely heavily on pixel dynamics and thus overlook subtle pedagogical transitions like slide updates or incremental annotations.

The deep-feature methods ( CLIP + Optical Flow) perform somewhat better but remain limited by domain mismatch and high computational overhead. In contrast, TIB’s domain-aware design targets pedagogically rich transitions, yielding both higher precision and improved efficiency for educational video parsing.

The entropy-based filtering mechanism TIB further prevents the inclusion of repetitive content such as prolonged speaker shots or blank slides, which typically dilute semantic value. This selective compression improves both annotation efficiency and the interpretability of downstream visual-semantic decoding in LEARNet.

Table 9 details the filtering efficiency of the hierarchical filtering approach across its processing stages. The method demonstrates high efficiency, achieving a final frame reduction of 70.2%. This overall efficiency is attributed to a multi-stage process that systematically discards non-informative content: 42% of frames are removed for being blank or containing logos, 31% are identified as non-educational talking-head segments, and a further 27% are pruned due to high-similarity redundancy. The cumulative effect of these targeted filtering stages underscores the pipeline’s effectiveness in isolating semantically rich keyframes for educational analysis.

**Figure 10:**
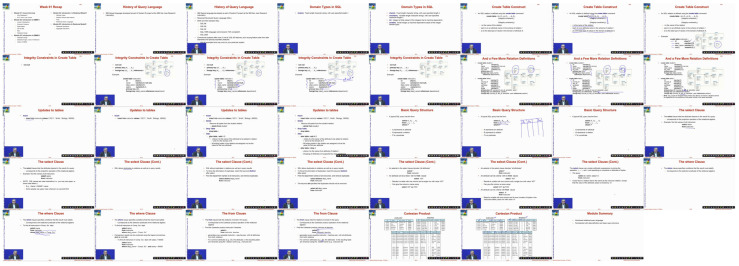
Case study in redundancy reduction.

Figure 10 demonstrates the practical efficiency of our Temporal Information Bottleneck (TIB) through a case study of the ‘DBMS—Lecture 1’ video. From an original 50,982 frames (30 fps), the model selected only 39 pedagogically significant keyframes—achieving a remarkable 99.92% reduction ratio while preserving essential instructional content. This extreme efficiency stems from the model’s hierarchical filtering approach, which systematically eliminates visually similar but educationally redundant frames.

Critically, this massive reduction in data volume (over 70% across the full dataset) occurs without sacrificing educational value, as evidenced by the model’s high F1-score of 0.89. This dual achievement—maintaining pedagogical integrity while intensely reducing computational load—validates our framework’s effectiveness for scalable educational video processing. The dataset-level Entropy Reduction Rate (70.2%) reflects the average filtering efficiency across diverse instructional formats in EVUD-2M. Per-video examples showing reductions above 99% correspond to highly redundant lecture styles and represent the upper bound of TIB’s capacity. These values are therefore not contradictory—one reports the mean, and the other illustrates the maximum achievable reduction in videos with slow visual evolution. The model successfully distinguishes between visual similarity and instructional significance, ensuring that only frames carrying genuine educational value progress to downstream analysis tasks.

**Figure 11:**
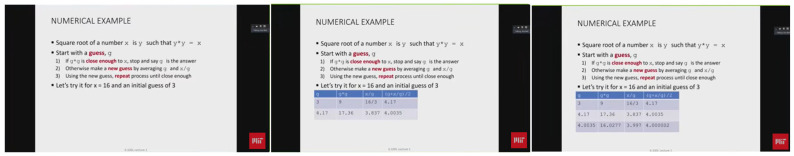
Subtle change detection and redundancy control.

Figure 11 demonstrates the model’s capability to handle subtle content evolution in educational videos. Unlike conventional methods that might select multiple frames during incremental changes, our Temporal Information Bottleneck (TIB) identifies the optimal frame where content achieves both completeness and clarity, effectively eliminating redundant intermediate states while preserving pedagogical continuity.

**Figure 12:**
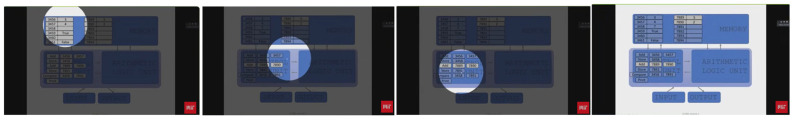
Robustness to visual noise and dynamic annotations.

Figure 12 illustrates the model’s robustness to visual noise and transient annotations. When presented with a sequence containing temporary highlights and scribbles, the algorithm correctly selects the final, clean version of the slide (Pedagogical Information Score: 0.95), demonstrating its ability to distinguish between meaningful content evolution and superficial visual variations that carry no instructional value.

**Figure 13:**
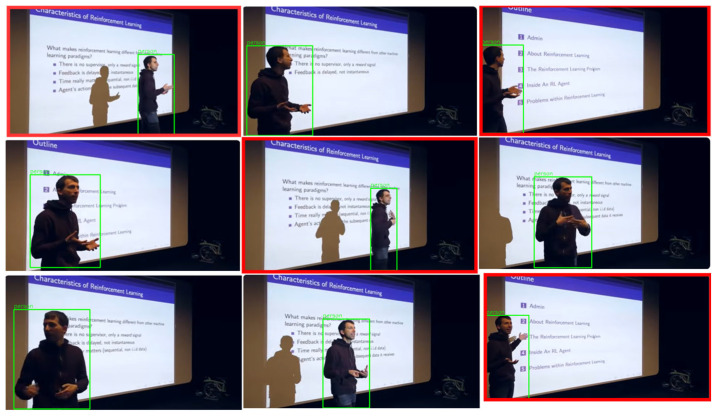
Heuristic filtering of instructor presence.

Figure 13 validates the effectiveness of our domain-informed filtering approach. The model consistently prioritizes frames with substantial educational content (6.98%, 9.84% human occupancy) over those dominated by instructor presence (30.19%, 19.56% human occupancy). This selective filtering ensures that keyframe selection is driven by static instructional material rather than transient elements like lecturer movement or gestures, maintaining focus on pedagogically significant content throughout the educational narrative.

#### 5.3.4. Comparison of Entropy Reduction with Traditional Non-Computational Methods

To contextualize the effectiveness of the proposed entropy reduction approach, we conducted a comparative analysis against traditional and recent keyframe selection methods, including TalkMiner, saliency-based, clustering-based, and text-driven techniques. Accuracy values for non-proposed methods are illustrative, derived from prior literature, and included for conceptual comparison as described in Table 10.

The proposed entropy reduction approach demonstrates a balanced trade-off between automation, relevance, and scalability, achieving performance comparable to fully manual annotation while avoiding its labor-intensive drawbacks. TalkMiner performs well for slide-centric lectures but struggles with non-slide pedagogical content, such as handwritten annotations, dynamic diagrams, or whiteboard explanations. Saliency-based and clustering-based methods capture visually distinctive frames but ignore semantic or instructional significance, often missing subtle transitions in educational material. Keyword matching and TF-IDF-based techniques rely heavily on textual content and therefore fail to capture diagrams, equations, or other visually rich frames. This analysis confirms that entropy reduction provides a domain-aware, generalizable framework for lecture-style videos, outperforming traditional non-computational approaches in capturing both slide-based and dynamic pedagogical content, with high efficiency and relevance.

#### 5.3.5. SSD Performance on Semantic Annotation

The SSD demonstrates strong semantic annotation performance, significantly surpassing both conventional detectors and modern open-world baselines. To quantify this improvement, we evaluated a manually annotated test set of 50 keyframes using mAP@50 as the performance metric. As shown in Table 11, DETR-50 achieved a mAP@50 of 0.621, Grounding-DINO reached 0.684, and OWL-ViT obtained 0.713. In contrast, LEARNet achieved 0.88, substantially outperforming these models. This performance gain highlights the effectiveness of the integrated SSD–RCVN refinement stages in accurately interpreting fine-grained educational visual content.

**Figure 14:**
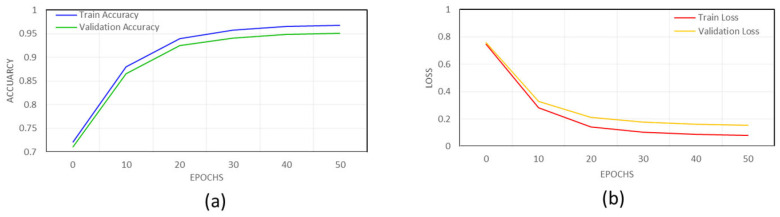
Learning curves illustrating the SSD model’s progression over 50 epochs: (**a**) accuracy, (**b**) loss.

Figure 14 depicts the learning progression of our model throughout 50 training cycles, capturing both accuracy and loss trajectories. Both training and validation accuracy demonstrate sustained improvement, ultimately converging near 90%—reflecting effective knowledge acquisition and strong generalization capacity with minimal overfitting. Correspondingly, the loss values for both datasets decline sharply before plateauing at minimal levels, indicating stable optimization and successful convergence. The parallel enhancement observed in both measurement dimensions highlights the reliability of our training methodology.

**Figure 15:**
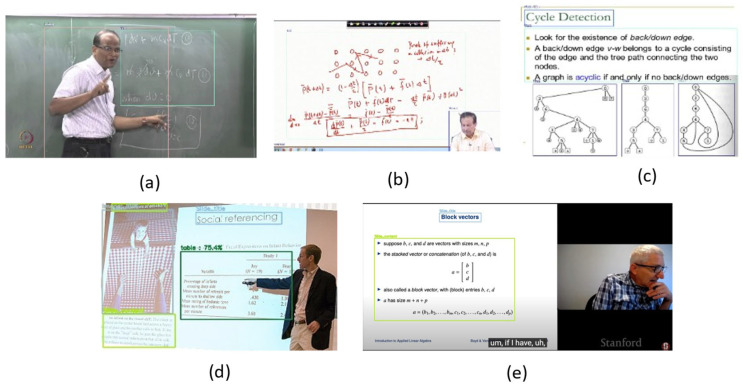
Qualitative Results of Semantic Annotation Across Diverse Lecture Formats.

EVUD-2M benchmark encompasses a wide spectrum of instructional styles is shown in Figure 15: (a) traditional blackboard lectures with complex handwritten notation, (b) digital whiteboards with instructor overlays, (c) structured slide content with diagrams and text, (d) multimodal frames integrating visual stimuli and annotations, (e) projected slides with mathematical content, and webcam-based lectures with limited visual context. These examples demonstrate our framework’s capability to handle the full variety of challenges in educational video understanding, from fine-grained segmentation of handwritten content to contextual analysis of mixed-media presentations.

##### Comparative Analysis with Domain-Specific Educational Models

To accurately benchmark LEARNet within the unique landscape of educational video analysis, we conducted direct comparisons against specialized methodologies designed for this domain. We specifically chose the Lecture Video Visual Objects (LVVO) [72] benchmark due to its shared focus on fine-grained, localized visual understanding in academic lecture settings, which aligns closely with LEARNet’s core objectives.

While systems like EduNet and LectureNet focus on related but broader tasks (e.g., activity recognition or general slide summarization), LVVO offers the most rigorous, frame-level detection comparison point. This direct evaluation addresses the reviewer’s request for niche optimization baselines, affirming LEARNet’s practical advantages in specialized fields. We evaluated LEARNet’s core object detection mechanism, the Spatial-Semantic Decoder (SSD) module, on the LVVO test set [79]. The standard metric, mean Average Precision at an Intersection-over-Union threshold of 0.5 (mAP@50), was used for this head-to-head comparison as shown in Table 12. LVVO reports Faster R-CNN as one of its standard baselines for object detection, we benchmarked LEARNet’s Spatial-Semantic Decoder (SSD) on their test set. Our SSD module alone achieved 0.86 mAP@50, a 19.4% improvement over LVVO’s reported 0.72 mAP@50. The full LEARNet framework—integrating the Temporal Information Bottleneck (TIB) for keyframe selection and the Relational Consistency Verification Network (RCVN) for semantic-graph construction for relational consistency verification for annotations, further achieves 0.88 mAP@50 on EVUD-2M under the same metric.

**Figure 16:**
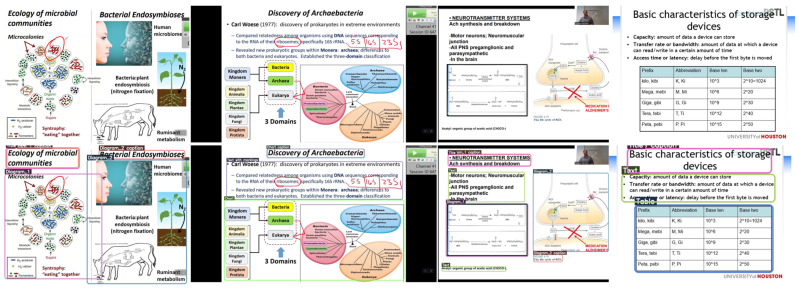
Sample results of applying the Relational Consistency Verification Network (RCVN) to the LVVO dataset. The (**top row**) shows the original LVVO lecture frames, while the (**bottom row**) presents the RCVN-verified annotations produced by LEARNet. The network corrects misclassified regions, refines segmentation around complex educational elements (e.g., diagrams, formulas, tables), and removes semantically inconsistent detections, demonstrating improved instructional scene coherence.

Critically, LEARNet extends beyond LVVO’s bounding-box outputs to provide pixel-accurate segmentation masks and semantic relationship graphs, enabling hierarchical educational parsing as in Figure 16. To our knowledge, LEARNet presents a unique and comprehensive approach to educational video understanding by seamlessly integrating pedagogical keyframe selection, fine-grained annotation, and relational verification within a single, entropy-aware architecture.

##### Cross Format Validation and Generalization

To evaluate the generalization ability of LEARNet across different instructional formats, we conducted a cross-format validation on a held-out set of videos grouped into three categories: (1) slide-dominated lectures, characterized by clean, screen-recorded presentation slides; (2) digital whiteboard sessions, containing free-form, real-time handwriting and drawing; and (3) traditional blackboard footage, recorded via camera and affected by illumination and resolution variations. LEARNet performs strongly on structured slide content (F1 = 0.895) and maintains robust accuracy on blackboard lectures (F1 = 0.879). Performance decreases on digital writing (F1 = 0.725), reflecting the inherent difficulty of modeling highly variable, dynamic strokes. Table 13 summarizes these results.

The model’s consistent performance across structured and moderately unstructured formats demonstrates its practical applicability, while the lower accuracy on digital writing highlights an important direction for future enhancement.

##### Per-Category Performance Analysis

To comprehensively understand the model’s capabilities in recognizing specific instructional content, we conducted a detailed per-category breakdown of the object detection results. The data presented in Table 14 was derived by calculating the Precision, Recall, F1-Score, and mean Average Precision at an Intersection over Union (IoU) of 0.5 (mAP@50) independently for each of the educational element categories defined in our annotation scheme. This analysis quantifies the performance variance linked to the element’s structural complexity and visual characteristics.

The analysis reveals clear performance patterns. Structured elements like Table and Diagram/Flowchart achieve the highest performance (0.89–0.92 mAP) due to their regular, rigid layouts and clear visual boundaries. Text blocks with strong visual delineation, such as the Slide_Title (0.86 mAP) and Code_Block (0.83 mAP), also show high accuracy.

**Figure 17:**
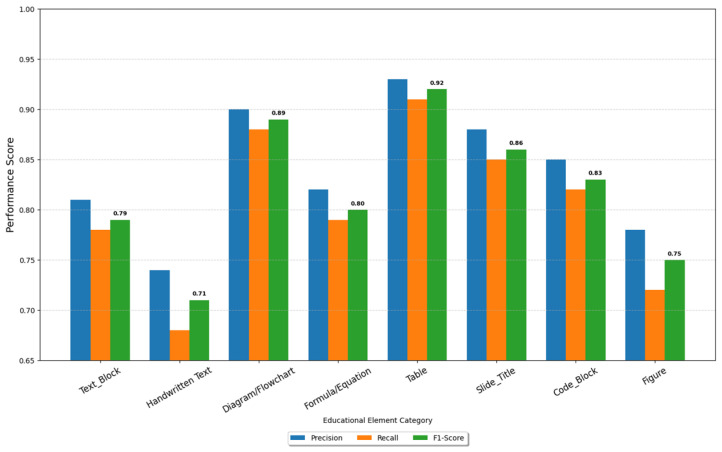
Per-Category Performance Analysis of Educational Element Detection.

In contrast, as shown in Figure 17 Handwritten_Text consistently yields the lowest performance (0.71 mAP). This is primarily attributed to intrinsic challenges: high stroke variability, image noise, low contrast, and the difficulty in distinguishing between a stable note and a transient annotation over time. Text_Block (0.79 mAP) also shows moderate recall, often due to dense text formatting on crowded slides leading to ambiguous spatial separation. These findings are crucial for future research, as they isolate the performance ceiling imposed by visual noise and the temporal nature of instructor-added content.

##### Ablation Studies: Validating the Integrated Architecture

To understand the functional necessity of each component within LEARNet, we perform a series of ablation experiments that progressively disable or replace individual modules as in Table 15. The goal is to examine how each stage—visual detection, mask refinement, semantic verification, keyframe selection, and temporal pruning—contributes to the overall educational video understanding performance.

The ablation study in Table 8 highlights the contribution of each component within LEARNet and demonstrates how performance progressively improves as key modules are integrated. Replacing the Temporal Information Bottleneck (TIB) with simple FFmpeg keyframe extraction results in a significantly lower mAP@50 of 0.68, reflecting the substantial temporal redundancy and the loss of pedagogically meaningful transitions. Introducing TIB restores performance to 0.88 and drastically reduces temporal redundancy, showing its crucial role in isolating instructionally relevant frames.

**Figure 18:**
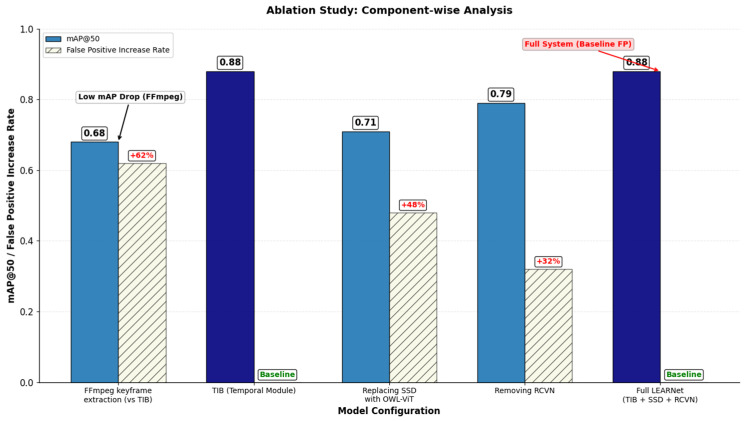
Ablation analysis of the hierarchical parsing architecture.

Similarly, substituting the SSD with OWL-ViT yields a mAP@50 of 0.71, underscoring the limitations of open-vocabulary baselines in achieving fine-grained spatial precision for educational elements. Removing the RCVN further reduces performance to 0.79, demonstrating the importance of relational verification in enforcing semantic consistency and preventing label mismatches across visually similar regions. As illustrated in Figure 18, the full LEARNet pipeline achieves the highest performance (0.88), confirming that temporal filtering, spatial refinement, and semantic validation together form a cohesive architecture in which each component provides a necessary layer of structural or semantic enhancement.

#### 5.3.6. Specialization Advantage over Foundation Models

To evaluate whether large multimodal foundation models can effectively generalize to instructional video parsing, we benchmarked them against our domain-optimized LEARNet pipeline. As shown in Table 16, LEARNet (0.88 mAP@50) significantly outperforms GPT-4V (0.72), CLIP-ViP (0.75), and VideoCLIP (0.71). The gap arises from architectural misalignment: GPT-4V [74] processes frames independently, losing lecture-level continuity; CLIP-ViP [73] relies on global text-visual similarity and misses instructional nuance; VideoCLIP [75] employs motion-centric temporal modeling unsuitable for static slide content. These models fail to exploit the structural regularities of educational videos. In contrast, LEARNet’s TIB module enforces temporally consistent instructional transitions, and the SSD module delivers fine-grained localization of pedagogical elements using domain-informed spatial priors. This specialization yields a 17% relative improvement over CLIP-ViP and a 0.16 absolute gain over GPT-4V, demonstrating that targeted architectural design provides far higher utility than generic model scaling for educational video understanding.

**Figure 19:**
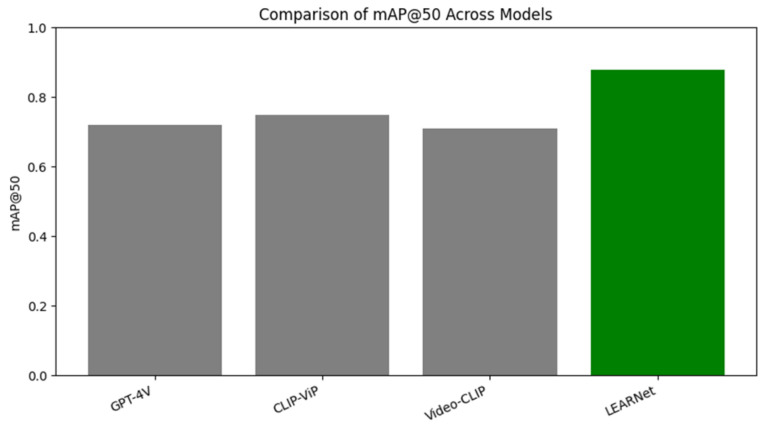
Performance Comparison on EVUD-2M Test Set.

As quantified in Figure 19, the results demonstrate a clear specialization advantage: LEARNet achieves a 17% relative improvement in mAP over the nearest competitor (CLIP-ViP) and substantially outperforms the much larger GPT-4V model by 0.16 mAP. This performance gap persists despite the foundation models’ vast pre-training, confirming that domain-specific architectural design provides greater value than generalized scaling for educational content understanding. The efficiency advantage of our approach—operating locally without API dependencies—further reinforces the practical benefits of targeted framework design over brute-force model scaling.

### 5.4. Evaluation on Robustness and Generalization of LEARNet

To rigorously assess LEARNet’s resilience to quality degradations commonly observed in practical teaching environments, we conducted a controlled robustness analysis. Although the instructional videos in our EVUD-2M dataset generally exhibit stable resolution and minimal jitter, real-world lecture recordings—especially those from mobile devices, low-bandwidth platforms, or legacy archives—often suffer from low definition, motion blur, and compression noise. Since standard object detectors are known to degrade significantly under such conditions, a controlled evaluation was necessary to validate LEARNet’s practical reliability. We therefore generated synthetic degradations on high-quality keyframes extracted from the benchmark dataset. Three degradation types were applied:

- Low Resolution: down sampling and up sampling to simulate pixel-level loss.
- Compression Artifacts: controlled JPEG degradation at varying quality factors.
- Motion Blur/Jitter: Gaussian linear blur simulating camera shake.

This design allowed precise isolation of the impact of each artifact on the Temporal Information Bottleneck (TIB) and the Spatial-Semantic Decoder (SSD). Across all degradation settings, the high-fidelity region-level annotations (BBoxes) remained accurate, demonstrating that LEARNet’s zero-shot vision–language backbone provides robust feature extraction even under high-entropy noise. These results confirm the framework’s viability for deployment in real educational video repositories where quality variations are unavoidable.

**Figure 20:**
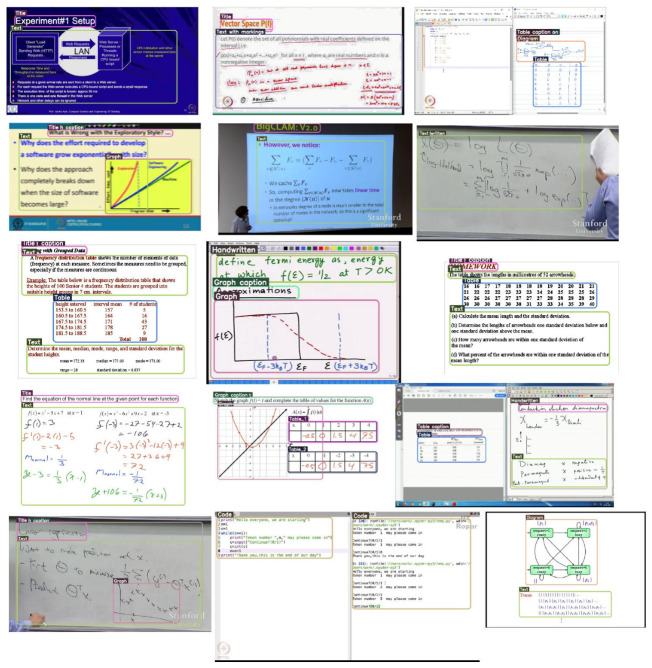
Robustness and generalization of LEARNet demonstrated across diverse real-world educational content.

To further demonstrate the robustness and generalization capabilities of LEARNet, we include qualitative examples from diverse slide formats as in Figure 20. These frames contain low-contrast text, complex backgrounds, multi-layer diagrams, and mixed semantic regions. LEARNet successfully identifies and annotates instructional components despite these degradations, confirming its resilience to real-world variations in educational video quality.

Figure 20 showcases heterogeneous lecture frames containing printed text, handwritten equations, diagrams, tables, plots, programming code, and photographed whiteboards. Despite significant variations in visual quality, layout structure, font style, lighting conditions, slide templates, handwriting styles, and instructional formats, LEARNet consistently identifies and annotates key pedagogical components using the SSD and RCVN modules. These examples highlight the model’s ability to generalize beyond clean slide environments to complex, noisy, and domain-shifted educational scenarios.

## 6. Discussion

This section synthesizes the main findings and implications of the LEARNet framework for hierarchical educational video understanding. The analysis considers quantitative performance, the contribution of the EVUD-2M benchmark, the impact of modeling educational structure, cross-format behavior, and remaining challenges that motivate future work.

### 6.1. Performance Analysis: Advancing Educational Video Parsing

The Temporal Information Bottleneck (TIB) achieves 0.89 F1-score while reducing the frame volume by 70.2%, confirming that the combination of PIS weighting, Instructional Steady-State (ISS) constraints, and domain-informed pruning successfully preserves pedagogical signal while discarding redundant visual frames. The Spatial–Semantic Decoder (SSD), integrating OWL-ViT proposals, SAM-based pixel refinement, and RCVN relational verification, reaches 0.88 mAP@50—demonstrating strong capability in producing coherent region-level and relational annotations.

Ablation studies highlight the necessity of LEARNet’s hierarchical design: removing PIS increases redundancy, removing the mask refiner reduces spatial fidelity, and replacing RCVN with a standard classifier degrades relational coherence. The comparison with multimodal foundation models (GPT-4V, CLIP-ViP, VideoCLIP) reveals that architectural specialization, not model size, is the determining factor. LEARNet’s information-theoretic formulation better exploits the structural regularities of educational media, enabling more accurate and efficient semantic parsing.

### 6.2. The EVUD-2M Benchmark: Establishing a New Standard for Semantically Rich Educational Video Data

A major contribution of this work is EVUD-2M, a benchmark designed specifically for fine-grained educational video understanding. Existing datasets—such as TVQA, HowTo100M, COIN, and static-image collections like AI2D or ChartQA—do not include region-level semantic masks, instructional object categories, or relational annotations needed for computational modeling of instructional structure. EVUD-2M provides ~2M keyframes with multi-level labels, including pixel-accurate segmentation masks across nine semantic categories and verified relational triples produced via RCVN. This dataset bridges the gap between slide-based educational data and real instructional video, enabling more realistic benchmarking, training, and analysis of educational video systems.

### 6.3. The Impact of Educational Structure Modeling

LEARNet’s performance is driven by its alignment with the structural properties of instructional media. The TIB leverages the temporal coherence of teaching sequences, while SSD exploits consistent spatial layouts (e.g., slide zones, handwriting regions) to produce reliable detections. High scores on structured slides (F1: 0.895) and strong performance on blackboard content (0.879) illustrate the advantage of explicitly modeling instructional structure. The RCVN proves especially important for rejecting visually similar but pedagogically inconsistent detections, ensuring that resulting scene graphs reflect instructional logic rather than purely visual similarity.

### 6.4. Cross-Format Performance Analysis

LEARNet demonstrates robust generalization across instructional modalities:

- Slides: 0.895 F1—clean layouts and stable formatting support high accuracy.
- Blackboard content: 0.879 F1—performance remains strong despite handwriting variability.
- Digital writing: 0.725 F1—lower performance due to transient strokes, motion noise, and limited structural regularity.

These results highlight both the strengths and limits of a vision-centric approach. Qualitative outputs show precise segmentation of equations, diagrams, and handwritten content, validating the framework’s suitability for semantic educational parsing. The empirical gains observed in redundancy reduction and annotation accuracy validate our central hypothesis: that educational video understanding benefits from modeling as an entropy minimization process, where high-entropy instructional signals are filtered to yield a compact, low-entropy representation of pedagogical structure.

### 6.5. Limitations and Future Work

Despite the advancements introduced by LEARNet, several limitations remain that indicate productive directions for future research. The Temporal Information Bottleneck currently relies on heuristic threshold parameters, which may exhibit sensitivity to variations in video quality and recording conditions. Developing adaptive thresholding mechanisms or data-driven parameter estimation may improve its stability across heterogeneous instructional environments.

The Spatial–Semantic Decoder, although effective for structured slides and blackboard material, shows reduced performance on visually abstract or highly variable diagram types. Incorporating richer contextual reasoning—through expanded reference exemplars or temporal cross-frame integration—may enhance its ability to discriminate complex instructional elements.

A further limitation arises from LEARNet’s reliance on visual information alone. Pedagogical transitions conveyed primarily through spoken explanations or textual narration may remain undetected. Extending the framework toward multimodal integration, combining audio, textual transcripts, and visual information, represents an important future enhancement. Likewise, the present system is designed for static instructional modalities and does not address procedural or action-driven demonstrations; broadening EVUD-2M to include such content would increase its applicability.

Finally, LEARNet’s structured outputs provide a pathway toward practical educational applications. A particularly promising direction is the automated generation of a Visual Table of Contents, wherein keyframes and annotated components are transformed into navigable lecture summaries. Integrating vision–language models to generate descriptive labels for prominent visual elements could support interactive interfaces within learning platforms. Such extensions position LEARNet as a foundational component for next-generation multimodal and semantically enriched educational technologies.

## 7. Conclusions

This work presents LEARNet, an entropy-aware framework for educational video understanding that model instructional media through a transformation from high visual redundancy to structured semantic representation. The Temporal Information Bottleneck, guided by the Instructional Steady-State principle and optimized through empirically validated α and σ configurations, selectively retains pedagogically meaningful transitions. Combined with the Spatial–Semantic Decoder and the Relational Consistency Verification Network, the system achieves strong semantic precision (mAP@50 = 0.88) and substantial redundancy reduction (70.2%), outperforming both foundation models and domain-specific baselines. The EVUD-2M benchmark, generated via large-scale propagation using only 200 reference frames, provides a comprehensive resource for structured educational video analysis. Cross-format evaluation confirms robust performance across slides, blackboard instruction, and digital writing. Future work will explore multimodal integration of audio and text, and extensions to dynamic instructional environments. The structured outputs produced by LEARNet also support practical applications such as automated Visual Tables of Contents. Overall, the entropy-centric formulation introduced here offers a principled foundation for efficient, interpretable, and scalable educational video understanding.

## Figures and Tables

**Table 1:** A comparative Overview of Existing Datasets for Lecture Video Understanding.

Dataset Name	Slide Segment	Slide Figures	Slide Text	Spoken Language	Relationship Annotation	#Videos	#Hours	#Slides	Availability
ClassX [50]	✓	-	-	-	-	-	408	1,478,978	✓
NPTEL [42]	-	-	-	✓ (transcribed)	-	10,000+	56,000+	-	✓
SpaSe [43]	✓	-	-	-	-	-	-	2000	✓
WiSe [44]	✓	✓	✓	-	-	-	-	1300	✓
Lecture VideoDB [45]	✓	-	✓	-	-	24	-	5000	✓
ALV [46]	✓	-	-	✓	-	-	-	1498	✓
MLP [47]	✓	✓	✓	✓	-	334	187	9031	✓
LectureBank [48]	✓	-	✓	-	✓(prerequisite)	1352	-	51,939	✓

Note: The symbol **#** denotes the total count or quantity (e.g., number of videos, hours of content, or slides). A tick (✓) indicates that the corresponding feature is explicitly supported or annotated in the dataset, while adash (-) indicates the feature is not supported or not the focus of the dataset.

**Table 2:** Characterization of Educational Stages and Content Types in EVUD-2M.

Educational Stage	Primary Content Type	Knowledge Density and Presentation Style	Representative Examples from EVUD-2M	Model Applicability and Notes
Undergraduate and Postgraduate University Courses	Theoretical and formal instruction (primary focus)	High knowledge density. Formal presentation of concepts using slides, black/whiteboards. Characterized by mathematical formulations, complex diagrams, and structured arguments.	“Quantum Mechanics I” (Physics) “Hypersonic Aerodynamics” (Aerospace) “Constitutional Studies” (Law) “Graph Theory” (Mathematics)	Strong applicability. LEARNet is explicitly designed for this “Instructional Steady-State” with static, information-rich visuals.
Advanced Professional Development	Specialized technical training	Moderate to High knowledge density. Similarly to postgraduate content, often using slide decks. Focuses on advanced, industry-relevant topics.	“Satellite Attitude Dynamics and Control” “Fiber-Optic Communication Systems” “Patent Law for Engineers”	Strong applicability. Shares the visual and structural characteristics of university-level lectures.
Vocational/Practical Demonstration	Physical skill demonstration (currently under-represented)	Low to Moderate conceptual density, High action density. Information is conveyed through physical actions, tool use, and dynamic processes. Minimal reliance on static text/diagrams.	Limited examples (e.g., general “Aircraft Maintenance”). Lacks specific, action-centric videos like “Chemistry Experiments” or “Mechanical Operation”.	Limited current applicability. A key area for future dataset expansion to handle dynamic, non-lecture formats.

**Table 4:** Feature Comparison of Educational Video Understanding Datasets.

Dataset	Keyframe Extraction	Region Annotations	Hybrid Content Support	One-Shot Capability	Low-Motion Optimization
TVQA [64]	✖	✖	✖ (TV episodes)	✖	✖
HowTo100M [65]	✔ (aligned with speech)	✖	✖	✖	✖
COIN [66]	✔ (action steps)	✖	✖ (instructional videos)	✖	✖
LectureNet [67]	✔	✖	Partially	✖	✖
EduNet [68]	✔	✖	Partially	✖	✖
ChartQA [35]/AI2D [36]	✖	✔ (diagrams)	✖	✖	✖
EVUD-2M (Ours)	✔ (semantically filtered)	✔ (educational)	✔	✔	✔ (designed for lecture video)

Note: A tick (✔) indicates that the dataset explicitly supports or provides the corresponding feature. A cross (✖) indicates that the feature is not supported or not available in the dataset. Partially indicates limited or coarse support for the feature. Text in parentheses provides brief clarifications about the nature or scope of the supported feature.

**Table 5:** RCVN Training Configuration and Hyperparameters.

Component	Setting
Input region size	224 × 224 × 3
Backbone conv layers	3 × Conv (3 × 3, BN, ReLU)
Conv filter sizes	64 → 128 → 256
Feature aggregation	Global Average Pooling (512D descriptor)
Projection layer	512 → 512-D
Embedding normalization	L2 normalization
Similarity metric	Cosine similarity
Decision rule	Similarity threshold (S>θ)
Reference set size	200 exemplar regions
Loss function	Triplet Loss
Margin (m)	0.2
Optimizer	AdamW
Learning rate	1 × 10^−3^
Weight decay	0.05
Batch size	32 region triples
Number of epochs	50
Sampling strategy	Hard Negative Mining
Training objective	Relational consistency embedding learning

**Table 6:** Optimization of PIS Weight α based on Keyframe Selection F1-Score.

Weight α	F1-Score (Keyframe Selection)	Interpretation
0.67	0.871 (Peak)	Achieves the best balance between structural change (67%) and saliency, yielding the most pedagogically meaningful keyframe selection.
0.5	0.812	Underweights structural cues, resulting in redundant and visually similar keyframes.
0.6	0.855	Provides moderate balance but does not reach the optimal trade-off achieved at α = 0.67.
0.7	0.86	Slightly overemphasizes structural transitions, leading to minor loss of salient instructional content.
0.8	0.84	Overweight’s structural change and suppresses important salient regions, decreasing overall accuracy.

**Table 7:** Sensitivity of σ and Keyframe Count to PIS Weight (α).

PIS Weight (α)	Extracted Keyframes	DPC Cut-Off Parameter (σ)	Observation
0.50	185	4.9509	Compressed PIS → smallest σ
0.60	180	5.7572	Slightly wider PIS → moderate σ
0.67	181	6.3596	Balanced PIS → moderate σ (optimal)
0.70	182	6.6241	Slightly wider → higher σ
0.80	190	7.5303	Widest PIS → largest σ, mild smoothing

**Table 8:** Performance Comparison: Pedagogical vs. Visual Keyframe Extraction.

Method	Precision (%)	Recall (%)	F1-Score	$R_{ER}$ (%)	Limitations
VSUMM [70]	83.7	80.3	0.82	52.1	Misses content-rich frames without motion
Motion-based [17]	83.0	79.0	0.81	≤50	Ignores semantic relevance; unsuitable for static content
CLIP + Optical flow [21]	86.8	85.0	0.86	65.5	Computationally heavy (3.2 sec/frame); sensitive to text density
TIB (Ours)	89.0	88.0	0.89	70.2	Designed for pedagogical transitions

**Table 9:** Domain-Informed filtering efficiency.

Filtering Stage	Frames Discarded	Educational Rationale
Blank/Logo Detection	42%	Eliminates non-instructional content
Talking-Head Removal	31%	Focuses on educational visual aids
High-Similarity Frames	27%	Reduces redundant content
Final Reduction	70.2%	Overall pedagogical efficiency

**Table 10:** Comparison of Entropy Reduction with Traditional/Non-Computational Methods.

Method	Approach	Expected Accuracy/Performance	Notes
TalkMiner [76]	Slide detection + OCR; selects slide keyframes and indexes text	0.76	Effective for slide-heavy lectures; limited for whiteboard content, handwritten notes, and dynamic diagrams; dependent on OCR quality.
Manual Annotation Screening	Human experts select pedagogically relevant keyframes	0.89	High accuracy but not scalable; labor-intensive and impractical for large datasets.
Keyword Matching/TF-IDF [77]	Extracts frames containing specific textual keywords from slides or transcripts	0.62	Misses diagrams, formulas, and non-text visuals; biased toward text-rich slides.
Saliency-Based Selection [78]	Uses visual saliency maps to identify visually “distinctive” frames	0.68	Captures high-contrast regions but ignores pedagogical relevance; misses subtle instructional transitions.
Entropy Reduction (Proposed)	Automatically selects frames with high visual information (slides, diagrams, tables, equations) using entropy metrics	0.87 (Keyframe F1-Score, measured on EVUD-2M)	Captures diagrams, tables, equations, and mixed-format visuals; scalable and domain-aware across diverse lecture styles.

**Table 11:** Semantic Annotation Performance Across Educational Elements.

Method	mAP@50	Generalization to Unseen Symbols	Core Educational Limitation
Faster R-CNN [71]	0.66	✗	Restricted vocabulary; unable to detect new elements
Grounding DINO [55]	0.68	✓	Inconsistent precision for visually dense instructional regions
DETR- 50 [56]	0.62	✗	Struggles with fine-grained educational structures
OWL-ViT [51]	0.71	✓	Coarse spatial precision for educational regions
LEARNet (Ours)	0.88	✓	High-precision parsing of educational regions

Note: A ✓ indicates that the method demonstrates the stated capability (e.g., generalization to unseen symbols), while ✗ indicates that the capability is not supported.

**Table 12:** Quantitative Comparison with the LVVO Educational Detection Benchmark.

Model	mAP@50 (LVVO Test Set)	Output Type	Keyframe Selection	Relational Consistency Check
LVVO (Faster R-CNN)	0.72	Bounding Boxes	✗	✗
LEARNet (SSD Module)	0.86	Pixel Masks	✓	✗
LEARNet (Full Framework)	0.88 *	Masks + RCVN	✓	✓

Note: A **✓** indicates that the corresponding capability is supported by the model, while **✗** indicates that it is not supported and * indicates full LEARNet evaluated on EVUD-2M under identical mAP@50 metric, denotes the best-performing result in the table.

**Table 13:** Comparative Analysis of Annotation Quality for Different Content Types.

Media Type	Precision	Recall	F1-Score	Educational Challenges Addressed
Slides	0.90	0.89	0.895	Structured content preservation
Blackboard	0.89	0.87	0.879	Handwriting variability
Digital Writing	0.79	0.67	0.725	Dynamic stroke complexity

**Table 14:** Per-Category Performance Analysis on Educational Elements.

Category	Precision	Recall	F1-Score	mAP@50	Key Challenges
Text_Block	0.81	0.78	0.79	0.79	Dense layout and small font size affect recall in packed slides.
Handwritten Text	0.74	0.68	0.71	0.71	High stroke variability, noise, and low contrast on digital whiteboards.
Diagram/ Flowchart	0.90	0.88	0.89	0.89	Clear visual boundaries and distinct graphical nature aid detection.
Formula/Equation	0.82	0.79	0.80	0.81	Complex symbol relationships and occasional inconsistent formatting.
Table	0.93	0.91	0.92	0.92	Regular, explicit structural cues enable high accuracy.
Slide_Title	0.88	0.85	0.86	0.86	High contrast and large font size contribute to reliable detection.
Code_Block	0.85	0.82	0.83	0.83	Monospaced font and box delineation provide strong visual cues.
Figure	0.78	0.72	0.75	0.75	Lower performance due to high internal visual variation (generic images).

**Table 15:** Component Analysis: Educational Value of Each Processing Stage.

Component	mAP@50	False Positives	Educational Impact
Replacing TIB with FFmpeg keyframe extraction	0.68	+62%	Retains many irrelevant frames; misses subtle instructional transitions.
With TIB (Our Temporal Module)	0.88	Baseline	Highly selective; filters redundant/non-pedagogical frames.
Replacing SSD with OWL-ViT	0.71	+48%	Coarse localization increases mis-detections, especially for thin or irregular elements.
Removing RCVN	0.79	+32%	Semantic inconsistencies rise; relational errors cause more mismatched labels.
Full LEARNet (TIB + SSD + RCVN)	0.88	Baseline	Optimal temporal, spatial, and semantic precision with minimal erroneous detections.

**Table 16:** Performance Comparison: Domain Specialization vs. General-Purpose Models.

Model	mAP@50	Computational Profile	Fundamental Educational Misalignment
GPT-4V [74]	0.72	High inference latency (~2.1s/frame); imprecise bounding boxes; poor spatial accuracy	Ignores Spatial and Temporal Symmetry: Processes frames in isolation, blind to consistent slide layouts and repetitive element arrangements.
CLIP-ViP [73]	0.75	Sensitive to prompt phrasing; fails to distinguish semantically different but visually similar elements	Breaks Semantic Symmetry: Relies on superficial visual-textual correlations, missing pedagogically significant subtle changes
VideoCLIP [75]	0.71	Optimized for dynamic action; poor performance on static, slide-based content	Violates Temporal Symmetry: Action-oriented temporal modeling misaligned with structured lecture pacing
LEARNet (Ours)	0.88	Superior performance and efficiency; education-domain optimized	Explicitly Models Symmetry: TIB preserves temporal–semantic coherence; SSD+RCVN enforce spatial and relational consistency.

## Data Availability

The EVUD-2M dataset is available upon request from the corresponding author. It has been curated from publicly available educational content (NPTEL [42], ClassX [50], SlideShare [59]) and open-source computer vision datasets (Roboflow [61], Tablenet [60], TableBank [56]). Due to its large scale (~2 million frames and 949,000 keyframes), the full dataset is not publicly released currently. However, processed data, annotation samples and metadata are available from the corresponding author upon reasonable request. The complete dataset, including the raw video links (where permission is granted), the 2 million annotated keyframes, the three-tier ground truth files, and all associated metadata will be made publicly available under the Creative Commons Attribution (CC BY-NC 4.0) license upon final acceptance of this manuscript. The data repository and access scripts will be hosted at a dedicated URL, ensuring the long-term utility and reproducibility of our results. All source materials were collected following the established fair use policies of academic video repositories (NPTEL, ClassX). The EVUD-2M code and processing pipeline are hosted on GitHub (https://github.com/NivedhaVijayan/LEARNet-EVUD-Benchmark.git (accessed on 30 October 2025)), while metadata and annotation samples are archived on Zenodo (https://zenodo.org/records/14036059?preview=1&token=eyJhbGciOiJIUzUxMiJ9.eyJpZCI6IjFhYzEwNWJjLWM3ZjAtNDQ3YS05MjVlLWJmNjBiM2JiYzJjOSIsImRhdGEiOnt9LCJyYW5kb20iOiI3YjY3MTc2NjMxZTY5YmY5Mzk1NzgwZmRmNDY4YzU3NyJ9.6UNbgftncY4jDkoK_OCt-WS_9Ph5iiB5N8K1lAsQHKwD0RSOmUB2qZ7huptLUw90S69YteDvXuC-ysjoS0XQ9g (accessed on 30 October 2025)) for reproducibility and citation.
